# Supplementary material for: Validating a novel driving simulation-based MWT against the standard MWT in an OSA-cohort challenged by CPAP-withdrawal (DS-MWT2) – Protocol for a monocentric, controlled, randomized, crossover trial
Source: PLoS One. 2026 Jul 9;21(7):e0341997. doi: 10.1371/journal.pone.0341997 (PMC13349176; doi:10.1371/journal.pone.0341997)
Supplement: S2 File — (DOCX) [file pone.0341997.s002.docx]

**DS-MWT2 – Validating a novel driving simulation-based MWT against the standard MWT in an OSA-cohort challenged by CPAP-withdrawal – a monocentric, controlled, randomized, crossover trial**

Study Type: Other Clinical Trial according to ClinO, Chapter 4

Risk Categorization: Risk category A

Study Registration: 1. Intended registration on clinicaltrials.gov

2. Intended registration in the FOPH portal SNCTP

Sponsor and Principal- Dr. rer. nat. Stefan Lakämper

Investigator: University of Zurich (UZH),

Institute for Forensic Medicine

Division of Traffic Medicine

Andreasstrasse 15

8050 Zurich

Switzerland

Investigator / Prüfärztin  Dr. med Kristina Keller

University of Zurich (UZH),

Institute for Forensic Medicine

Division of Traffic Medicine

Andreasstrasse 15

8050 Zurich

Switzerland

Co-Investigator PD Dr. med. Esther Irene Schwarz

University Hospital Zurich

Department of Pulmonology

Sleep Disorders Centre and Ventilation Unit

Raemistrasse 100

8091 Zurich

Switzerland

Investigated Intervention: Withdrawal of CPAP-treatment for ≥ 7d.

Protocol ID DS-MWT2

Version and Date: Version 1.2 (dated 10/03/2025)

**Protocol Signature Form**

| Study Title | DS-MWT2 – Validating a novel driving simulation-based MWT against the standard MWT in an OSAS-cohort challenged by CPAP-withdrawal – a monocentric, controlled, randomized, crossover trial |
| --- | --- |
| Study ID | SNCTP000006301 \| BASEC2024-01948  intended registration on clinicaltrials.gov |

The Sponsor-Investigator has approved the protocol version 1.3 (dated 08/07/2025) and confirms hereby to conduct the study according to the protocol, current version of the World Medical Association Declaration of Helsinki, and ICH-GCP guidelines as well as the local legally applicable requirements.

**Sponsor-Investigator:**

Name: Dr. rer. nat. Stefan Lakämper

Date: Signature:

**Investigator / verantwortliche Prüfperson**

Name: Dr. med. Kristina Keller

Date: Signature:

# Table of Contents

Table of Contents 4

GLOSSARY OF ABBREVIATIONS 5

1 STUDY SYNOPSIS 6

2 BACKGROUND and rationale 14

3 Study OBJECTIVES and Design 15

3.1 Hypothesis and primary objective 15

3.2 Primary and secondary endpoints 16

3.3 Study design 16

3.4. Study intervention 16

4 Study POPULATION and Study procedures 16

4.1 Inclusion and exclusion criteria, justification of study population 16

4.2 Recruitment, screening and informed consent procedure 17

4.3 Study procedures 18

4.4 Withdrawal and discontinuation 21

5 STATISTICS AND METHODOLOGY 22

5.1. Statistical analysis plan and sample size calculation 22

5.2. Feature Description

5.3. Handling of missing data and drop-outs 24

6 Regulatory Aspects AND SAFETY 25

6.1 Local regulations / Declaration of Helsinki 25

6.2 (Serious) Adverse Events and notification of safety and protective measures 25

6.3 (Periodic) safety reporting 26

6.4 Radiation 26

6.5 Pregnancy 27

6.6 Amendments 27

6.7 Notification and reporting upon completion, discontinuation or interruption of the study 27

6.8 Insurance 27

7 FURTHER Aspects 29

7.1 Overall ethical considerations 29

7.2 Risk-benefit assessment 29

8 Quality CONTROL AND Data protection 30

8.1 Quality measures 30

8.2 Data recording and source data 30

8.3 Confidentiality and coding 31

8.4 Retention and destruction of study data and biological material 32

9 MonitoriNg and Registration 32

10. Funding / Publication / declaration of Interest 32

11. REFERENCES 33

Appendix 1: Schedule of assessments 33

# GLOSSARY OF ABBREVIATIONS

*AE Adverse Event*

*ASR Annual Safety Report*

*BASEC Business Administration System for Ethical Committees*

*CRF Case Report Form*

*CTCAE Common Terminology Criteria for Adverse Events*

*FADP Federal Act on Data Protection (in German: DSG, in French: LPD, in Italian: LPD)*

*eCRF electronic Case Report Form*

*FOPH Federal Office of Public Health*

*GCP Good Clinical Practice*

*HRA Human Research Act (in German: HFG, in French: LRH, in Italian: LRUm)*

*ICH International Conference on Harmonisation*

*ClinO Ordinance on Clinical Trials in Human Research (in German: KlinV, in French: OClin, in Italian: OSRUm)*

*SAE Serious Adverse Event*

*n.a. not applicable*

*EEG Electroencephalogram*

*ET Eye-tracking*

*DS driving simulation*

*M, MWT maintenance of wakefulness test*

*D, DS-MWT driving simulated maintenance of wakefulness test*

*IDS integrated driving score*

*SAFE Standardized Application for Fitness to Drive Evaluations*

*SDLP standard deviation of lane position*

*AOI area of interest*

*V Visit*

*ESS Epworth Sleepiness scale*

*KSS Karolinska Sleepiness scale*

*EDS excessive daytime sleepiness*

*OSA Obstructive sleep apnea*

*CPAP continuous positive airway pressure*

*W CPAP withdrawal*

*C CPAP continuation*

*SA Sleep Accident*

*AASM American Academy of Sleep Medicine*

# STUDY SYNOPSIS

| **Sponsor / Sponsor-Investigator** | Dr. rer. nat. Stefan Lakämper  University of Zurich (UZH),  Institute for Forensic Medicine  Division of Traffic Medicine  Andreasstrasse 15  8050 Zurich  Switzerland  Mobile: +41 79 379 88 94  Office: +41 44 635 76 70  E-Mail: [stefan.lakaemper@irm.uzh.ch](mailto:stefan.lakaemper@irm.uzh.ch) |
| --- | --- |
| **Study Title** | DS-MWT2 – Validating a novel driving simulation-based MWT against the standard MWT in an OSAS-cohort challenged by CPAP-withdrawal – a monocentric, controlled, randomized, crossover trial |
| **Short Title / Study ID** | DS-MWT2 |
| **Protocol Version and Date** | Version 1.1 (dated 14/02/2025) |
| **Study Registration** | SNCTP000006301, clinicaltrails.gov intended |
| **Study Category and Rationale** | Other Clinical Trial, Risk category A |
| **Background and Rationale** | In brief, the proposed study will evaluate a recently proposed naturalistic, driving simulation test (1) to identify and measure sleepiness behind the wheel, one of the most underestimated causes of road accidents. The proposed test offers higher ecological validity and might complement somnological tests that are standard, but rarely performed. Thus, the test might provide traffic medicine and sonologists with an effective tool, that can also directly covey the risks of excessive daytime sleepiness to drivers and thus, in combination, effectively aid in traffic medicine’s mandate to avoid preventable road fatalities (2).  Excessive daytime sleepiness (EDS) is a symptomatic condition resulting from too little or compromised sleep, caused by psychosocial stress (shiftwork, lifestyle) or medical conditions (obstructive sleep apnoea (OSA), narcolepsy). Driving with untreated EDS might lead to sleepy/drowsy driving and microsleep, which is considered to be one of the highest-ranking causes of road accidents. Sleepiness and its dangers on the wheel might subjectively not be registered by the affected drivers. Also, subjective sleepiness might not correlate with somnological measurements that are also crucial for legally determining the fitness to drive (FTD).  There exist a variety of partially complementary tools to evaluate the extent of EDS (1). Mean sleep latencies obtained in the maintenance of wakefulness tests (MWT) are widely, but not uniformly, considered to be one of the most objective measures to evaluate EDS, especially in the context of driving performance (1-4). However, there is inconsistent or insufficient evidence for MWTs to reliably predict the FTD in general, potentially as its result might be strongly influenced by motivation (5). Moreover, patients might not relate low mean MWT-latencies to their own and other’s risks in traffic.  A need for improved tools to measure EDS was formulated (6). It remains open, whether the MWT should be replaced or complemented by, for example, future road-side metabolomics-tests (7) detecting sleepiness in traffic or whether the MWT should be adapted to better convey a.) the risks of EDS in traffic and b.) its meaning for the determination of the FTD.  With these goals in mind we proposed furnishing the maintenance of wakefulness test with improved ecological validity to provide an improved tool for the assessment of the effect of excessive daytime sleepiness on the fitness to drive: we recently showed results from an exploratory feasibility study suggesting it to be well possible to transfer the MWT-paradigm to a driving simulator (DS) with high user acceptance (1). The published result’s implication and relevance was well received (2): the new test, DS-MWT, might complement somnological MWTs in pneumology and neurology. I might provide a naturalistic and relatable tool to determine EDS in traffic medicine, who is institutionally responsible for determining the FTD. This is also desirable, because prohibitively high cost - in time, money and instrumentation - often prevent a standard MWT in standard care of sleep-related medical conditions. Potentially, the use of the DS-MWT might help reduce the number of preventable road fatalities by more often identifying sleepy individuals before they get behind the wheel (2).  However, for this goal to achieve, it remains so far to be evaluated whether the latencies obtained in classical or simulation conditions are comparable and whether obtained latencies actually reflect other clinical parameters of EDS relating to underlying medical conditions, such as for example OSA. This represents a significant gap of evidence for both medical experts in pneumology and traffic medicine, but also for affected drivers. We will fill this gap by systematically comparing classical or simulation-based MWTs by means of their resulting latencies. In a within-study setup of 30 participants, we relate our experiments to a main medical comparator, a ≥7-day continuous positive airway pressure (CPAP)-withdrawal (W) and subsequent -resumption or continuation (C), respectively, in highly adherent OSA-patients (9). |
| **Risk / Benefit Assessment** | The participation in this study involves only minimal risks (category A)  The study procedures at each one of 7 visits consist of standardized and mostly non-invasive tests. Participants are under constant supervision.  In rare cases, the use of the driving simulator can lead to dizziness and discomfort (simulator sickness), but these quickly subside when leaving the simulator or completing the test.  EEG mounting and blood sampling using a peripheral venous donor catheter will be conducted by trained personnel. Hematoma from blood sampling and/or skin irritations from EEG electrodes may occur rarely, but these usually disappear without further complications. The collected full blood volume of 9 ml is generally considered harmless to health. There are no health risks in the sampling of urine, saliva, dried blood spots, and exhaled breath.  For the intervention “CPAP-withdrawal” (W), participants omit their overnight use of CPAP treatment at home for a ≥ 7-day period (typically holidays) and thus will have a reactivation of OSA. This intervention has been validated as a safe effective and well-controlled model to study the pathophysiological consequences of OSA (3, 4). At the end of the study, patients will resume their established long-term CPAP therapy. All patients will be primarily assessed by physicians from the Sleep Disorders Center on whether a CPAP-withdrawal can cause them health risks. Only preselected patients with no risks to their health will be included in the study. Professional drivers will be excluded from the study.  Otherwise, participation in the study is not associated with any known specific risks.  There is no direct health benefit for the study participants.  Overall social and scientific benefits are described in section 7.1. |
| **Objective(s)** | (H_p,1_) Primary hypothesis: no statistically significant difference between MWT- and DS-MWT-latencies (primary outcome measure).  (O_p_) Primary objective: We will determine whether or not – and if yes, to which extent – there is a statistically significant difference between MWT- and DS-MWT-latencies  The primary hypothesis will be tested solely on determined mean latencies and separately from additional, secondary and exploratory analyzes.  (H_s_) Additional, secondary, exploratory hypotheses: There are statistically significant correlations between conditions CPAP-withdrawal vs. CPAP-treatment to secondary test – and performance parameters (see 3.2 secondary outcome measures, see 5.2 Feature Description)  Additional, secondary, exploratory objectives are:   - (O_s-1_) We will explore, whether or not – and if yes, which and to which extent – the **test-sequence** affects MWT- and DS-MWT-latencies and other test- and performance parameters. - (O_s-2_) We will explore, whether or not – and if yes, which and to which extent – **driving parameters** such as, for example, standard deviation of lateral position, speed, distance and steering wheel angle, SDLP, SDS, SDD and SDSW or integrated driving scores, IDS, correlate to the prior treatment or withdrawal of CPAP and the outcomes of the Epworth Sleepiness Scale (ESS) and Karolinska Sleepiness Scale (KSS) (1). Additionally, time-course-effects of, for example, SDLP will be analyzed using either repeated measures-ANOVA of 10 4-min-long modules comprising the test drive or using paired t-tests of the first and last driven module (1). - (O_s-3_) We will explore, whether or not – and if yes, which and to which extent – **eye tracking parameters** such as, for example, blink rate, blink duration, eye openness, gaze direction, gaze duration and areas of interests (5, 6), correlate to the prior treatment or withdrawal of CPAP in the driving simulation. - (O_s-4_) We will explore, whether or not – and if yes, which and to which extent – **EEG features** that potentially indicate sleepiness, such as, for example, microsleep-like episodes, MSE, or individual signatures of MSE and the sleep/wake transition (7), correlate to the prior treatment or withdrawal of CPAP and the outcomes of the ESS and KSS. - (O_s-5_) We will explore, whether or not – and if yes, which and to which extent – **repeated @home-PSG-measurements** reveal night-to-night variability of OSA-parameters (8) such as, for example, AHI, ODI, ¢SpO2, t<90, and how they correlate to other outcome parameters. CPAP data will be used to control for the adherence to the instructions and for OSA--activation control. - (O_s-6_) We will explore, whether or not – and if yes, which and to which extent – previously identified potential (proteomic) **biomarkers for sleepiness (9-11) in non-invasive samples** such as saliva, exhaled breath, dried bloodspots (12), correlate to the prior treatment or withdrawal of CPAP and the outcomes of the ESS and KSS. |
| **Endpoint(s)** | (Ep) Primary endpoint: significant difference between MWT- and DS-MWT-latencies.  Additional, secondary, exploratory endpoints are  (Es-1) significant differences in driving parameters  (Es-2) significant differences in ET- and EEG-features  (Es-3) significant differences in @home-PSG-recordings and test sequence  (Es-4) significant correlation to biomarkers for sleepiness in saliva and/or exhaled breath  Endpoints are understood as significant changes in outcome measures (see 5.2 for Feature Description for further details)  See 5.1.2 for statistical analysis plan |
| **Study Design** | This study is a monocentric, controlled, within-subjects, randomized, cross-over clinical trial on a cohort of OSAS patients. Two equivalent arms explore the influence of the test sequence per starting condition. Neither participants nor observers can be fully blinded.  The study visits (V1-V7, see Figure 1) will be attended by all participants.  Allocation to the starting condition (W or C) and one of two study arms (DM or MD) determining measurement sequence is required and will be randomized (lottery).  Study schedule, intervention and measurement sequence prevent complete observer- or participant-blinding.  *A priori,* we do not allocate or stratify by study population characteristics, i.e., according to sex, age, medication, dosage, duration of treatment etc. We will, however, evaluate *post hoc*, whether outcomes are influenced by these factors. |
| **Statistical Considerations** | There is no dedicated statistician other than the investigators involved.    sample size calculation (see chapter 5.1.1)  The total sample size is N_total_ = 54, consisting of the intervention group N_I_ = 36 and a healthy comparison group N_H_ = 18.  statistical analysis plan (see chapter 5.1.2)    The primary hypothesis will be tested solely on determined mean latencies and separately from additional, secondary and exploratory analyzes. For the primary hypothesis, we will employ a mixed model ANOVA or generalized linear mixed model. All analyzes will be considered significant when p<0.05.  All secondary variables will be analyzed post-hoc for hypothesis-generation rather than formal testing. Collected secondary parameters will be analyzed *post hoc* individually with respect to the experimental condition (D/M) and the participants state (CPAP-treatment or withdrawal). If group-level differences exist we will conduct multiple comparison tests. Time-course-effects of, for example, SDLP are intended to be analyzed using repeated measures-ANOVA between 10 4-min-long modules comprising the test drive or using paired t-tests of the first and last driven module. Integrated Driving Scores (IDS, Z-scores) will be calculated after logistic regression of driving parameters. For secondary parameter-based models, we will first use linear mixed models and stepwise testing will be based on biological/plausibility justification rather than purely statistical significance. Akaike Information Criterion (AIC) and k-fold cross-validation will be used to for prediction models of sleep onset/sleep accidents on the level of individual 40 min runs.  Significant findings from exploratory analyzes will be interpreted cautiously and will most likely require independent validation in future studies.  We cannot reliably aim at uncovering gender differences, but will include sex or gender in any model for control.  The statistical software R, Python, and GraphPad Prism will be used.  Any deviation from the original statistical plan will be described and justified in the final trial report. We intend to complete the study with N_total_ = 54, except recruitment success is (deemed) prohibitive within the projected study duration (LPLV 30/04/2027, see section 4.3). |
| **Inclusion- / Exclusion Criteria** | Inclusion criteria (OSA patients):   - adult driver - diagnosed OSA, - established CPAP-treatment regime, highly adherent and compliant within the last 6 months (>5h, >80% of days), - at impaired eyesight with more than +/- 5 diopter or astigmatism, contact lenses are required (for eye tracking)   Inclusion criteria (healthy comparison croup):   - adult driver - no declared psychiatric disorders, - no declared sleep-related diagnosis, - at impaired eyesight with more than +/- 5 diopter or astigmatism, contact lenses are required (for eye tracking)   Exclusion criteria (for both groups):   - sensibility to motion sickness (kinetosis, dizziness, etc. in 5 min screening drive), - professional drivers (if working during the study period), - inability to understand the study procedure for linguistic or cognitive reasons.   Based on the inclusion criteria and the preselected pool for recruitment, we will not include any vulnerable participants as defined to the Swiss HRA (chapter 3, i.e. children, adolescents, adults lacking capacity, or prisoners). However, participation in the experimental procedure does not pose any additional risks for pregnant women, neither for patients, healthy adults or the unborn child. Therefore, the participation of pregnant individuals is possible and a pregnancy test is not required. |
| **Number of Participants with Rationale** | The total sample size is N_total_ = 54, consisting of the intervention group N_I_ = 36 and a healthy comparison group N_H_ = 18.  For sample size calculation (see chapter 5.1.1)  For the intervention group, we will recruit from a highly adherent pool of patients with OSA treated in the Sleep Disorders Center and Pulmonary Division, University Hospital of Zurich, Zurich, Switzerland. For the comparison group (healthy adults), we will recruit from the public.  The restricted pool for the study population has the advantage that it is highly coherent in treatment and supervision. Members of the pool are already preselected by the referring partner institution (Sleep Disorders Center) for not having a.) substantial comorbidities (such as major affective, somatic or neurological disorders) or cognitive deficits and b.) any additional risks caused by the intervention, i.e. CPAP-withdrawal. |
| **Study Intervention** | Withdrawal of CPAP-treatment for ≥ 7d |
| **Control Intervention** | n.a. |
| **Study procedures** | **Table 1.** Overall overview of the visits. Timing between visits 1 and 2 as well as 4 and 5 will have to be ≥ 7 days but can vary, visits 2-4 and 5-7 will have to take place on subsequent days (see also Figure 1).  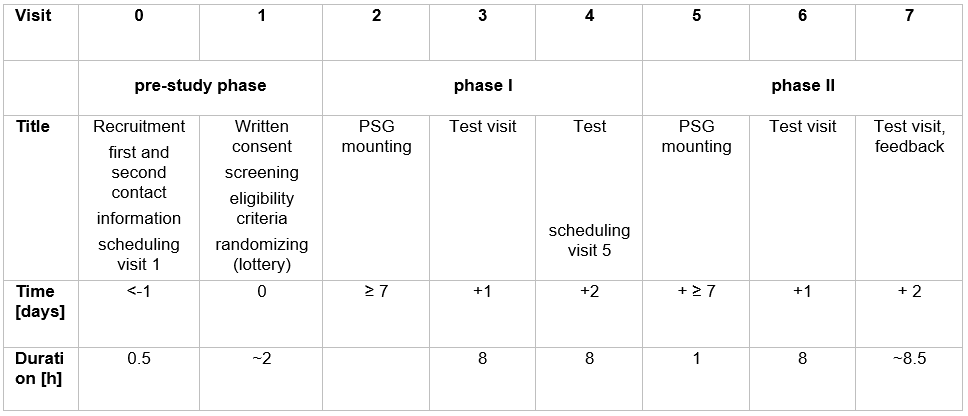  **Table 1.** Schedule and summary of each study visit including all planned interventions, tests, questionnaires, and biological sampling. * = only after written consent.  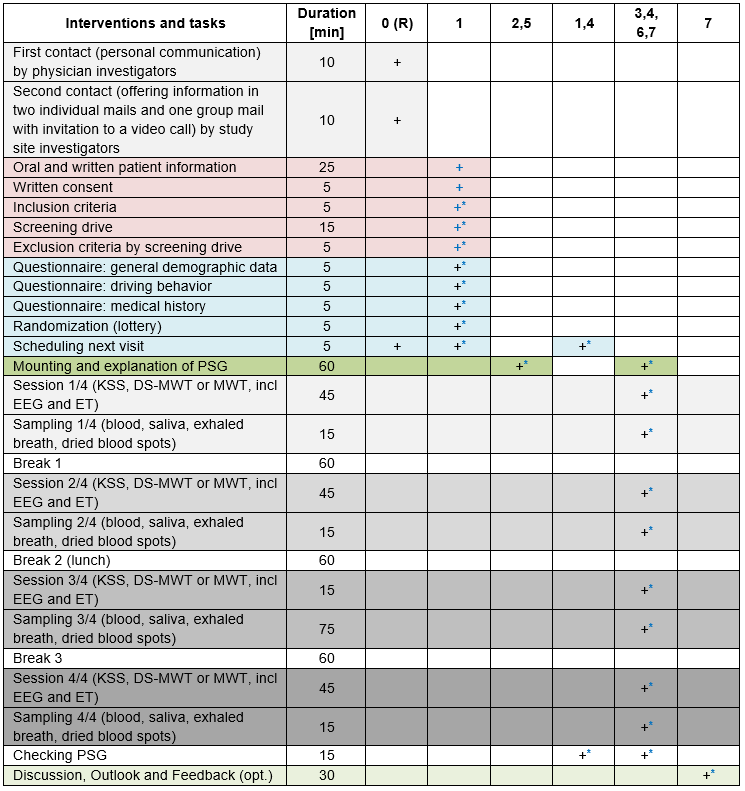 |
| **Study Duration and Schedule** | planned 01/05/2025 First-Participant-in (FPFV)  (anticipated date of the screening/enrolment of the first patient)  planned 30/04/2027 Last-Participant-Out (LPLV)  (anticipated date of the last participant’s final visit  planned 30/04/2028 published manuscript / submission of final report |
| **Investigator(s)** | Dr. rer. nat. Stefan Lakämper  University of Zurich (UZH),  Institute for Forensic Medicine  Division of Traffic Medicine  Andreasstrasse 15  8050 Zurich  Switzerland  Dr. med. Kristina Keller  University of Zurich (UZH),  Institute for Forensic Medicine  Division of Traffic Medicine  Andreasstrasse 15  8050 Zurich  Switzerland  PD Dr. med. Esther Irene Schwarz  University Hospital Zurich  Department of Pulmonology  Sleep Disorders Centre and Ventilation Unit  Raemistrasse 100  8091 Zurich  Switzerland |
| **Study Center(s)** | University of Zurich (UZH),  Institute for Forensic Medicine  Division of Traffic Medicine  Andreasstrasse 15  8050 Zurich  Switzerland |
| **Data privacy** | Investigators are responsible for ensuring that all study-related information and data collected are kept strictly confidential. Any disclosure of information to people who are not directly involved in the study must be approved by the owner of the data. Data collection, disclosure, archiving and analysis of personal data within the study are carried out strictly in accordance with the applicable Swiss data protection regulations. The prerequisite for this is the voluntary consent of the trial participants, which is given by signing the declaration of consent before participating in the clinical trial. The data collected as part of this clinical trial can be viewed for verification or inspection by monitors and the independent Ethics Committee.  No genetic data will be collected in this exploratory study, thus not encoded.  Biological material in this study is not identified by participant name but by a unique participant number. Biological material is appropriately stored in a restricted area only accessible to the authorized personnel.  The safely stored biological data at the Institute for Forensic Medicine is protected by several layers of access restrictions at the site. Further instructions will be documented to ensure traceability by the study team. The maintenance of a cooling system will be ensured by the technical staff of the Institute for Forensic Medicine. |
| **Ethical consideration** | Study procedure, sample-taking and intervention are non-invasive and associated with minimal risks and burdens. The procedures are mainly standardized and participants are already used to conduct CPAP holidays. The only side-effect of the study procedure might be skin irritations (from EEG) or symptoms linked to kinetosis during the driving simulation, but will rapidly subside. The study design in section 3.3 is appropriate for the study population. The study team is adequate for this small exploratory study. Hence, ethical considerations with respect to risks are minimal.  The overall social and scientific value is given as an increased fraction of the global population suffers from sleep deficits and a considerable fraction of traffic accidents (15-30%) can be traced back to symptoms of excessive daytime sleepiness (EDS) affecting drivers. Therefore, studying the effect of CPAP-withdrawal (as often performed voluntarily during holidays) will be informative for traffic safety. The information gained is useful for OSAS-diagnosed patients and practitioners. Having a naturalistic read-out (virtually “real-life” driving performance) in comparing the DS-MWT to the classical MWT will also help the applied sciences to serve as a control for developing improved, understandable and potentially simplified diagnostic tools for determining the extent of EDS, especially in the context of assessing the fitness to drive. Hence, the benefits of the proposed study are ethically justified.  We will not include any vulnerable participants. |
| **GCP Statement** | This study will be conducted in compliance with the protocol, the current version of the Declaration of Helsinki, the ICH-GCP, the HRA as well as other locally relevant legal and regulatory requirements. |

#

# BACKGROUND and rationale

In brief, the proposed study will evaluate a recently proposed naturalistic, driving simulation test (1) to identify and measure sleepiness behind the wheel, one of the most underestimated causes of road accidents. The proposed test offers higher ecological validity and might complement somnological tests that are standard, but rarely performed. Thus, the test might provide traffic medicine and sonologists with an effective tool, that can also directly covey the risks of excessive daytime sleepiness to drivers and thus, in combination, effectively aid in traffic medicine’s mandate to avoid preventable road fatalities (2).

Excessive daytime sleepiness (EDS) is a symptomatic condition resulting from too little or compromised sleep, caused by psychosocial stress (shiftwork, lifestyle) or medical conditions (obstructive sleep apnoea (OSA), narcolepsy). Driving with untreated EDS might lead to sleepy/drowsy driving and microsleep, which is considered to be one of the highest-ranking causes of road accidents. Sleepiness and its dangers on the wheel might subjectively not be registered by the affected drivers. Also, subjective sleepiness might not correlate with somnological measurements that are also crucial for legally determining the fitness to drive (FTD).

There exist a variety of partially complementary tools to evaluate the extent of EDS (1). Mean sleep latencies obtained in the maintenance of wakefulness tests (MWT) are widely, but not uniformly, considered to be one of the most objective measures to evaluate EDS, especially in the context of driving performance (1-4). However, there is inconsistent or insufficient evidence for MWTs to reliably predict the FTD in general, potentially as its result might be strongly influenced by motivation (5). Moreover, patients might not relate low mean MWT-latencies to their own and other’s risks in traffic.

A need for improved tools to measure EDS was formulated (6). It remains open, whether the MWT should be replaced or complemented by, for example, future road-side metabolomics-tests (7) detecting sleepiness in traffic or whether the MWT should be adapted to better convey a.) the risks of EDS in traffic and b.) its meaning for the determination of the FTD.

With these goals in mind we proposed furnishing the maintenance of wakefulness test with improved ecological validity to provide an improved tool for the assessment of the effect of excessive daytime sleepiness on the fitness to drive: we recently showed results from an exploratory feasibility study suggesting it to be well possible to transfer the MWT-paradigm to a driving simulator (DS) with high user acceptance (1). The published result’s implication and relevance was well received (2): the new test, DS-MWT, might complement somnological MWTs in pneumology and neurology. I might provide a naturalistic and relatable tool to determine EDS in traffic medicine, who is institutionally responsible for determining the FTD. This is also desirable, because prohibitively high cost - in time, money and instrumentation - often prevent a standard MWT in standard care of sleep-related medical conditions. Potentially, the use of the DS-MWT might help reduce the number of preventable road fatalities by more often identifying sleepy individuals before they get behind the wheel (2).

However, for this goal to achieve, it remains so far to be evaluated whether the latencies obtained in classical or simulation conditions are comparable and whether obtained latencies actually reflect other clinical parameters of EDS relating to underlying medical conditions, such as for example OSA. This represents a significant gap of evidence for both medical experts in pneumology and traffic medicine, but also for affected drivers. We will fill this gap by systematically comparing classical or simulation-based MWTs by means of their resulting latencies. In a within-study setup of 30 participants, we relate our experiments to a main medical comparator, a ≥7-day continuous positive airway pressure (CPAP)-withdrawal (W) and subsequent -resumption or continuation (C), respectively, in highly adherent OSA-patients (9).

# Study OBJECTIVES and Design (Zielsetzung der Studie)

## 3.1 Primary Hypothesis and objective

(H_p,1_) Primary hypothesis: no statistically significant difference between MWT- and DS-MWT-latencies (primary outcome measure).

(O_p_) Primary objective: We will determine whether or not – and if yes, to which extent – there is a statistically significant difference between MWT- and DS-MWT-latencies

The primary hypothesis will be tested solely on determined mean latencies and separately from additional, secondary and exploratory analyzes.

(H_s_) Additional, secondary, exploratory hypotheses: There are statistically significant correlations between conditions CPAP-withdrawal vs. CPAP-treatment to secondary test- and performance parameters (see 3.2 secondary outcome measures, see 5.2 Feature Description)

Additional, secondary, exploratory objectives are:

- (O_s-1_) We will explore, whether or not – and if yes, which and to which extent – the **test-sequence** affects MWT- and DS-MWT-latencies and other test- and performance parameters.
- (O_s-2_) We will explore, whether or not – and if yes, which and to which extent – **driving parameters** such as, for example, standard deviation of lateral position, speed, distance and steering wheel angle, SDLP, SDS, SDD and SDSW or integrated driving scores, IDS, correlate to the prior treatment or withdrawal of CPAP and the outcomes of the Epworth Sleepiness Scale (ESS) and Karolinska Sleepiness Scale (KSS) (1). Additionally, time-course-effects of, for example, SDLP will be analyzed using either repeated measures-ANOVA of 10 4-min-long modules comprising the test drive or using paired t-tests of the first and last driven module (1).
- (O_s-3_) We will explore, whether or not – and if yes, which and to which extent – **eye tracking parameters** such as, for example, blink rate, blink duration, eye openness, gaze direction, gaze duration and areas of interests (5, 6), correlate to the prior treatment or withdrawal of CPAP in the driving simulation.
- (O_s-4_) We will explore, whether or not – and if yes, which and to which extent – **EEG-features** that potentially indicate sleepiness, such as, for example, microsleep-like episodes, MSE, or individual signatures of MSE and the sleep/wake transition (7), correlate to the prior treatment or withdrawal of CPAP and the outcomes of the ESS and KSS.
- (O_s-5_) We will explore, whether or not – and if yes, which and to which extent – **repeated @home-PSG-measurements** reveal night-to-night variability of OSA-parameters (8) such as, for example, AHI, ODI, ¢SpO2, t<90, and how they correlate to other outcome parameters. CPAP data will be used to control for the adherence to the instructions and for OSA--activation control.
- (O_s-6_) We will explore, whether or not – and if yes, which and to which extent – previously identified potential (proteomic) **biomarkers for sleepiness (9-11) in non-invasive samples** such as saliva, exhaled breath, dried bloodspots (12), correlate to the prior treatment or withdrawal of CPAP and the outcomes of the ESS and KSS.

## 3.2 Primary and secondary endpoints (outcomes, outcome measures, Zielgrössen)

Endpoints are understood as significant changes in outcome measures (see 5.2 for Feature Description for further details)

(Ep) Primary endpoint: significant difference between mean MWT- and DS-MWT-latencies.

Additional, secondary, exploratory endpoints are

(Es-1) significant differences in driving parameters

(Es-2) significant differences in ET- and EEG-features

(Es-3) significant differences in @home-PSG-recordings and test sequence

(Es-4) significant correlation to biomarkers for sleepiness in saliva and/or exhaled breath

See 5.1.2 for statistical analysis plan

## 3.3 Study design

This study is a monocentric, controlled, within-subjects, randomized, cross-over clinical trial on a cohort of OSAS patients. Two equivalent arms explore the influence of the test sequence per starting condition. Neither participants nor observers can be fully blinded.

Allocation to the starting condition (W or C) and one of two study arms (DM or MD) determining measurement sequence is required and will be randomized (lottery).

*A priori,* we do not allocate or stratify by study population characteristics, i.e., according to sex, age, medication, dosage, duration of treatment etc. We will, however, evaluate *post hoc*, whether outcomes are influenced by these factors.

## 3.4. Study intervention

Intervention: Withdrawal of CPAP-treatment for ≥ 7d

# Study POPULATION and Study procedures

## 4.1 Inclusion and exclusion criteria, justification of study population

The total sample size is N_total_ = 54, consisting of the intervention group N_I_ = 36 and a healthy comparison group N_H_ = 18.

For the intervention group, we will recruit from a highly adherent pool of patients with OSA treated in the Sleep Disorders Center and Pulmonary Division, University Hospital of Zurich, Zurich, Switzerland. For the comparison group (healthy adults), we will recruit from the public.

The restricted pool for the study population has the advantage that it is highly coherent in treatment and supervision. Members of the pool are already preselected by the referring partner institution (Sleep Disorders Center) for not having a.) substantial comorbidities (such as major affective, somatic or neurological disorders) or cognitive deficits and b.) any additional risks caused by the intervention, i.e. CPAP-withdrawal.

We will not be able to, at this point, ensure gender balance, which is due to the much higher prevalence of OSA in the male population. While there is the chance of having a balanced pool, a retrospective stratification seems out of scope due to the sample size and the between-patient variability of the outcome measures in the intervention group.

Inclusion criteria (OSA patients):

- adult driver
- diagnosed OSA,
- established CPAP-treatment regime, highly adherent and compliant within the last 6 months (>5h, >80% of days),
- at impaired eyesight with more than +/- 5 diopter or astigmatism, contact lenses are required (for eye tracking)

Inclusion criteria (healthy comparison croup):

- adult driver
- no declared psychiatric disorders,
- no declared sleep-related diagnosis,
- at impaired eyesight with more than +/- 5 diopter or astigmatism, contact lenses are required (for eye tracking)

Exclusion criteria (for both groups):

- sensibility to motion sickness (kinetosis, dizziness, etc. in 5 min screening drive),
- professional drivers (if working during the study period),
- inability to understand the study procedure for linguistic or cognitive reasons.

Based on the inclusion criteria and the preselected pool for recruitment, we will not include any vulnerable participants as defined to the Swiss HRA (chapter 3, i.e. children, adolescents, adults lacking capacity, or prisoners). However, participation in the experimental procedure does not pose any additional risks for pregnant women, neither for patients, healthy adults or the unborn child. Therefore, the participation of pregnant individuals is possible and a pregnancy test is not required.

## 4.2 Recruitment, screening and informed consent procedure

For the intervention group, we will recruit patients with OSA from a highly adherent preselected pool treated in the Sleep Disorders Center and Pulmonary Division, University Hospital of Zurich, Zurich, Switzerland. For the comparison group, we will recruit from the public.

Recruitment towards and up until inclusion will be accomplished by personal communication by phone, email, in a video call (optional) or in person at the study site.

First contact will be offered by a flyer containing contact information to the investigators.

Investigators will offer to explain details of the study either by phone, email, or a video call (optional) to the participants (either individually or in groups). If participants are interested, a first on-site visit is scheduled.

Investigators will explain to each participant the nature of the study, its purpose, the procedures involved, the expected duration, the potential risks and benefits and any discomfort it may entail.

Each participant will be informed that participation in the study is voluntary and that he or she may withdraw from the study at any time and that withdrawal of consent will not affect his or her subsequent medical assistance and treatment.

All participants for the study will be provided with a participant information sheet and a consent form describing the study and providing sufficient information for participants to make an informed decision about their participation in the study. Enough time as much as the participant desires will be given to decide whether to participate or not.

The formal consent of a participant, using the approved consent form, will be obtained before the participant is submitted to any study procedure.

The consent form will be signed and dated by the sponsor-investigator or his designee at the same time as the participant signs. A copy of the signed informed consent will be given to the study participant. The consent form will be retained as part of the study records.

The informed consent process will be documented in the patient file and any discrepancy to the process described in the protocol will be explained. The informed consent will be obtained from the participant at the first study visit before biological samples of blood and urine are collected and which obtained data will be used for further studies.

A 5-min-long screening drive will evaluate the only exclusion criterion (kinetosis, dizziness) and ensure a full understanding of the following task.

Participants will have no direct benefit from the study. As participation in a research project with no expected direct benefit may be appropriately remunerated, there will be a financial compensation. However, participants indirectly benefit form repeated measures of their sleep quality (PSG) that could otherwise not be performed in standard care situations. In addition, OSA patients indirectly benefit from getting to know their individual effects upon CPAP-withdrawal. Upon request, the collected information will be available for their further personal use for all participants.

According to the [Leitfaden Finanzielle Zuwendungen](https://swissethics.ch/assets/pos_papiere_leitfaden/entschaedigungen_pkl_d.pdf), Abschnitt 2 Modell b, we aim to compensate participation with **CHF 500 for the completed study**. An ethical guideline concerning the remuneration of participants is also available on [www.swissethics.ch](http://www.swissethics.ch).

Here we discussed two models for compensation:

Model A: strictly time-proportional, participants receive ca 12.00 CHF/hour spent at the study site.

Model B: content-proportional, participants receive 80 CHF if only visits 2 and 3 are completed, 180 CHF if visits including 4 are completed, 230 CHF if visit 5 including overnight PSG is completed, 350 CHF if visits including visit 6 are completed and the full 500 CHF if all study visits are included. This reflects an hourly compensation increasing from 12 CHF to 35.71 CHF if averaged cumulated over time on task on measurements or from 12 CHF to 20 CHF if calculated individually by time on task per visit.

Model B reflects the potential of increased information gained per completed visit. In addition, calculated increasing hourly rates per individual time on task reflect typical compensations without forcing undesired motivational aspects within the cohort (participation by financial benefit only). The compensation scheme reflects, that there is neither third-party financial benefit nor substantial risks to a participant’s health involved. It might be noted, that studies with similar intervention (3, 4) or higher compromise for the participants (12) did get no or lower compensation, respectively.

Weighing in attrition and stopping (partial reference) biases, we decided to compensate according to **model B**. A remaining risk of incomplete recruitment or increase drop-out within the allocated study period and, thus, potential effects on the power of the study, is acknowledged.

In any case, also the termination during or in between the study visits, participants will have to sign a receipt for the compensation.

## 4.3 Study procedures

The planned overall duration of the study is 24 months, with 01/05/2025 being First-Participant-in (FPFV, anticipated date of the screening/enrolment of the first patient) and 30/04/2027 being Last-Participant-Out (LPLV, anticipated date of the last participant’s final visit. A published manuscript or submission of the final report will be handed in by 30/04/2028.

Figures 1 and 2 as well as Tables 1 and 2 summarize all relevant procedures and study visits.


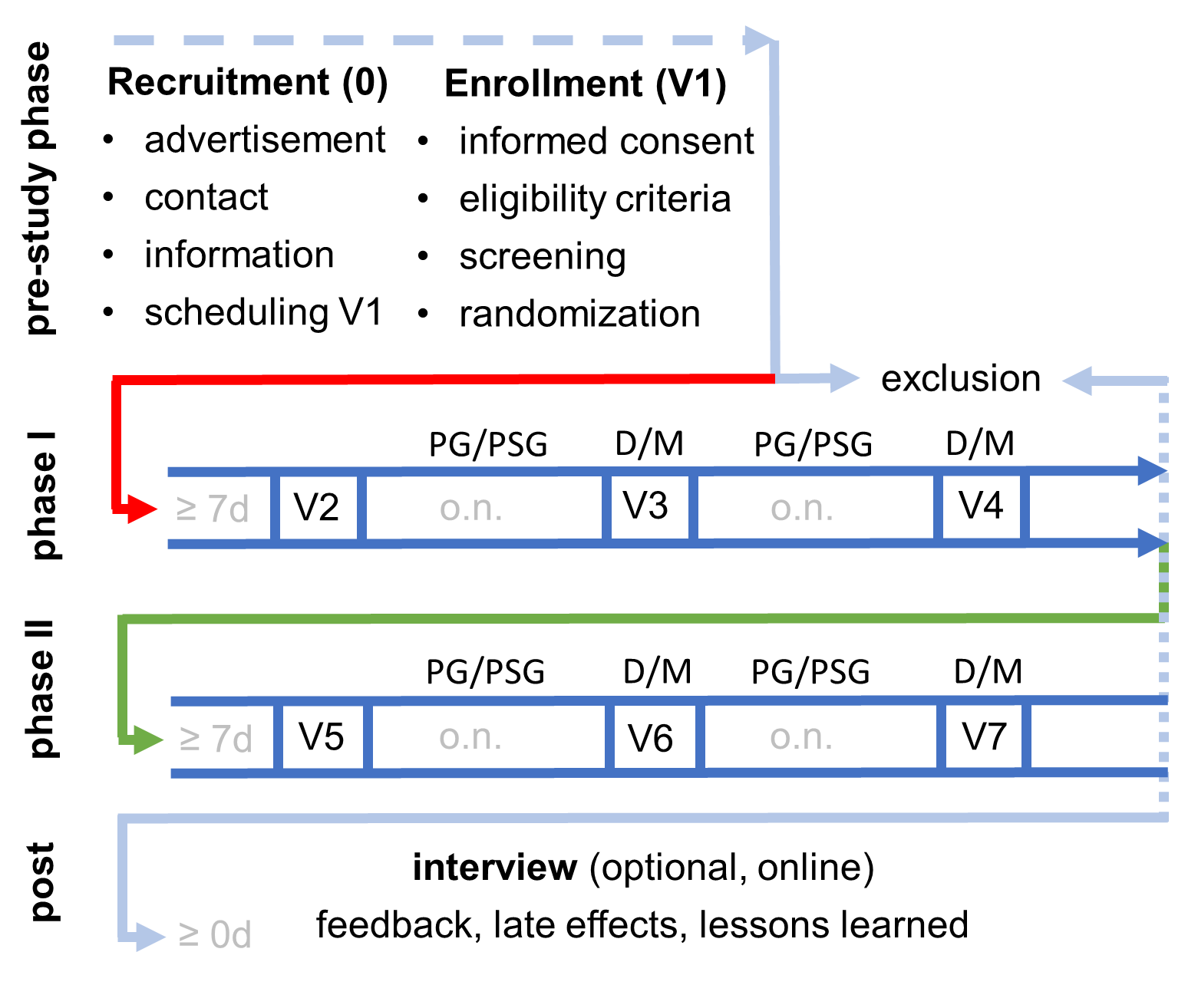


**Figure 1.** Overall course of the study. V = visit, PSG = @home-polysomnography, D = driving simulation-based maintenance of wakefulness test, M = maintenance of wakefulness test. Visit 1 comprises inclusion and a brief screening. Visit 2 and Visit 5 serve merely to mount PSGs.

Visit 1 comprises discussion and signing of the informed consent, inclusion, a brief screening drive to control for screening-based exclusion and the randomization lottery. In visit 2 and visit 5 the EEG-electrodes for @home-PSGs are mounted. PSGs are recorded overnight between visits 2-3, 3-4 and 5-6, 6-7 each. During visit 3, 4, 6 and 7 driving simulation-based maintenance of wakefulness tests (D) or maintenance of wakefulness tests (M) will be performed according to the respective study group, i.e. the randomized allocation of starting condition or sequence, respectively (see Figure 2). Randomization will be achieved by lottery during visit 1. Participants are invited to give feedback after the study (optionally personal or online).

Each driving simulation-based maintenance of wakefulness test (D) or maintenance of wakefulness test (M) in visits 3,4,6, and 7 comprises four 40-min-long sessions (also termed “runs”) with EEG-measurements including video, starting at 9 a.m. 11 a.m. 1 p.m. and 3 p.m.. Prior to each session, participants are asked to indicate their subjective level of sleepiness using the Karolinska Sleepiness Scale 8KSS). In each session, participants are asked to stay awake in a dark environment, either sitting still with open eyes without activation (M) or sitting still in a monotonous night-time car-following task in a driving simulator (D). Each driving simulation-based maintenance of wakefulness tests (D) includes eye tracking. Eye tracking will be included in maintenance of wakefulness tests (M), depending on availability of the instrument. Each session is followed by sample taking (blood, saliva, exhaled breath, dried blood spot). EEG-Electrodes are removed after visits 4 and 7, but checked throughout visits 3, 4, 6 and 7. An optional interview after the study phase is possible, if the patients wish to elaborate on the feedback, late effects, lessons learned, or the daily life effects they encountered after completion of all 7 study visits. Visit 1 requires an estimated presence of 1 hour. Visit 2 and 4 require an estimated presence of 1 hour. Visit 3, 4, 6, and 7 each require an estimated presence of 8 hours, with actual test durations of approximately 4 hours, i.e. four times 40-min log runs and 15 min-long sample taking.


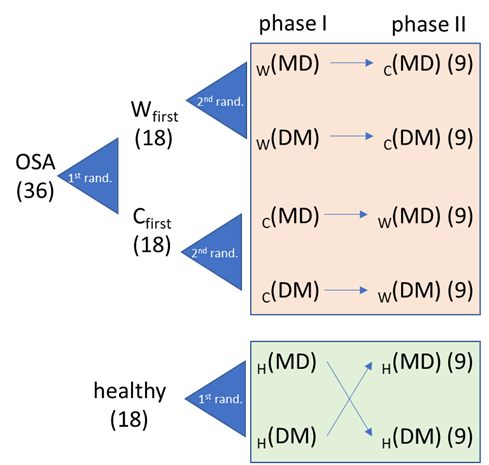


**Figure 2.** Overall randomization scheme for the intervention group and the comparison group. W_first_ = Group starting with the sequence in the condition “withdrawal”, i.e. the intervention. C_first_ = Group starting with the sequence in the condition “continuation”. D = driving simulation-based maintenance of wakefulness test, M = maintenance of wakefulness test.

**Table 1.** Overall overview of the visits. Timing between visits 1 and 2 as well as 4 and 5 will have to be ≥ 7 days but can vary, visits 2-4 and 5-7 will have to take place on subsequent days (see also Figure 1).

| **Visit** | **0** | **1** | **2** | **3** | **4** | **5** | **6** | **7** |
| --- | --- | --- | --- | --- | --- | --- | --- | --- |
|  | **pre-study phase** | | **phase I** | | | **phase II** | | |
| **Title** | Recruitment  first and second contact  information  scheduling visit 1 | Written consent  screening  eligibility criteria  randomizing (lottery) | PSG mounting | Test visit | Test  scheduling visit 5 | PSG mounting | Test visit | Test visit, feedback |
| **Time [days]** | <-1 | 0 | ≥ 7 | +1 | +2 | + ≥ 7 | +1 | + 2 |
| **Duration [h]** | 0.5 | ~1 | 1 | 8 | 8 | 1 | 8 | ~8.5 |

**Table 2.** Schedule and summary of each study visit including all planned interventions, tests, questionnaires, and biological sampling. ***** = only after written consent.

| **Interventions and tasks** | **Duration [min]** | **0 (R)** | **1** | **2,5** | **1,4** | **3,4,**  **6,7** | **7** |
| --- | --- | --- | --- | --- | --- | --- | --- |
| First contact (personal communication) by physician investigators | 10 | + |  |  |  |  |  |
| Second contact (offering information in two individual mails and one group mail with invitation to a video call) by study site investigators | 10 | + |  |  |  |  |  |
| Oral and written patient information | 25 |  | + |  |  |  |  |
| Written consent | 5 |  | + |  |  |  |  |
| Inclusion criteria | 5 |  | +**^*^** |  |  |  |  |
| Screening drive | 15 |  | +**^*^** |  |  |  |  |
| Exclusion criteria by screening drive | 5 |  | +**^*^** |  |  |  |  |
| Questionnaire: general demographic data | 5 |  | +**^*^** |  |  |  |  |
| Questionnaire: driving behavior | 5 |  | +**^*^** |  |  |  |  |
| Questionnaire: medical history | 5 |  | +**^*^** |  |  |  |  |
| Randomization (lottery) | 5 |  | +**^*^** |  |  |  |  |
| Scheduling next visit | 5 | + | +**^*^** |  | +**^*^** |  |  |
| Mounting and explanation of PSG | 60 |  |  | +**^*^** |  | +**^*^** |  |
| Session 1/4 (KSS, DS-MWT or MWT, incl EEG and ET) | 45 |  |  |  |  | +**^*^** |  |
| Sampling 1/4 (blood, saliva, exhaled breath, dried blood spots) | 15 |  |  |  |  | +**^*^** |  |
| Break 1 | 60 |  |  |  |  |  |  |
| Session 2/4 (KSS, DS-MWT or MWT, incl EEG and ET) | 45 |  |  |  |  | +**^*^** |  |
| Sampling 2/4 (blood, saliva, exhaled breath, dried blood spots) | 15 |  |  |  |  | +**^*^** |  |
| Break 2 (lunch) | 60 |  |  |  |  |  |  |
| Session 3/4 (KSS, DS-MWT or MWT, incl EEG and ET) | 15 |  |  |  |  | +**^*^** |  |
| Sampling 3/4 (blood, saliva, exhaled breath, dried blood spots) | 75 |  |  |  |  | +**^*^** |  |
| Break 3 | 60 |  |  |  |  |  |  |
| Session 4/4 (KSS, DS-MWT or MWT, incl EEG and ET) | 45 |  |  |  |  | +**^*^** |  |
| Sampling 4/4 (blood, saliva, exhaled breath, dried blood spots) | 15 |  |  |  |  | +**^*^** |  |
| Checking PSG | 15 |  |  |  | +**^*^** | +**^*^** |  |
| Discussion, Outlook and Feedback (opt.) | 30 |  |  |  |  |  | +**^*^** |

As all data are recorded automatically (Silab, SAFE, EEG, ET) and/or without interference from the investigator (questionnaires), we do not expect observer or sequence biases here. Any adherence/reporting bias (e.g. participants incorrectly report their medication status or by-consumption) is controlled for by blood sampling. Attrition and stopping (partial reference) biases are minimized by the compensation scheme.

## 4.4 Withdrawal and discontinuation

The participants take part in the study voluntarily and confirm their consent in writing. The participants have the right to discontinue the study at any time without giving reasons. The trial management may terminate the trial participation prematurely if a participant withdraws their consent if agreements in the subject information are not adhered to or if the eligibility criteria (see 4.1) are no longer met. Trial participants whose data cannot be used in full for the analyzes due to premature termination of the trial may be replaced. The data and samples collected up to that point can still be evaluated for individual analyzes. The data will be stored in accordance with section 8.2.

If the trial participation is terminated prematurely by the trial subject or the trial management, no additional final visit is planned. In the event of premature termination at any time during that visit, the participant can leave the trial center independently. If the participant feels unwell, a person of trust can be contacted to pick them up. If no person of trust can be reached or is available, they will be driven to their home address by a taxi company.

Should an adverse event (AE) occur during or after the study, the participant can contact the sponsor-investigator or the study physician, who will then take any necessary steps. At the discretion of the sponsor- investigator or study physician, a follow-up visit may be arranged with the subject.

# STATISTICS AND METHODOLOGY

## 5.1 Statistical analysis plan and sample size calculation

There is no dedicated statistician other than the investigators involved. However, a statistician will be available throughout the study and subsequent analyzes.

See section 3.1 for primary and secondary hypotheses.

### 5.1.1 Sample size calculation

The total sample size is N_total_ = 54, consisting of the intervention group N_I_ = 36 and a healthy comparison group N_H_ = 18. (see Figure 2)

Sample sizes N_I_ = 36 for the intervention group were estimated according to formula I considering the randomized crossover RCT study design of a cohort of OSA-patients randomized both to CPAP starting condition and measurement sequence resulting in 4 groups 4 (W-DM, W-MD, C-DM, C-MD, see Figure 2) in two phases and a mixed-model ANOVA analysis. Mixed-model ANOVA accounts for the fact that repeated measures form the same person are correlated, is able to handle any missing values more effectively and considers both fixes and random effects.


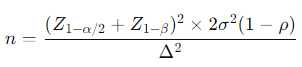


Formula I

adapted from (13)

- n = Required number of participants per group.
- Z_1−α/2_​ = Z-value corresponding to the chosen significance level (e.g., 1.645 for α = 0.1 two-sided) (14).
- Z_1−β_ = Z-value corresponding to the desired power, 1.28 for 90% power) (14).
- σ = Standard deviation of the measurements = 13.5 minutes (from Bonnet & Arand) (15)
- ρ = Intra-class correlation (ICC) or correlation between repeated measures within the same participant.
- Δ = Equivalence margin or effect size = 1 pooled SD = 13.5 minutes (from Bonnet & Arand) (15)

For the sample size calculations, we use a two-sided α = 0.1 assuming that a potential difference between M and D would have a clear direction and a maximal value (bounded test range) of 40 min for the test latency. When conducting an equivalence test, using a 0.1 two-sided alpha-level effectively matches the 0.05 significance level for each side of the distribution (one for each tail), aligning the rigor of the test with the traditional hypothesis test for no difference. This assumption holds, as the reference standard deviation of 13.5 min was obtained with an equivalently bounded test range. We employ a graphically extracted and pooled standard deviation form Bonnet & Arand (15) (Figure 2 baseline and wake, p 388), the only close-to test-retest data on MWTs available.

Similarly, sample sizes N_H_ = 18 for the **comparison group** (healthy) were estimated employing a crossover without retest RCT design for the test-retest approach. We find 9 for each of two groups for 90% (assuming a two-way ANOVA), respectively (Formula II), resulting in an overall sample size of 18 for two groups. This is despite using smaller 1SD (σ) = 5.9 minutes from normative data on healthy subjects from Banks et al. (16), but is explained by the lack of additional variability-reducing conditions as compared to the OSA-cohort.


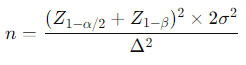


Formula II

adapted from (17)

### 5.1.2 Statistical analysis plan

The primary hypothesis will be tested solely on determined mean latencies and separately from additional, secondary and exploratory analyzes. For the primary hypothesis, we will employ a mixed model ANOVA or generalized linear mixed model. All analyzes will be considered significant when p<0.05.

All secondary variables will be analyzed post-hoc for hypothesis-generation rather than formal testing. Collected secondary parameters will be analyzed *post hoc* individually with respect to the experimental condition (D/M) and the participants state (CPAP-treatment or withdrawal). If group-level differences exist we will conduct multiple comparison tests. Time-course-effects of, for example, SDLP are intended to be analyzed using repeated measures-ANOVA between 10 4-min-long modules comprising the test drive or using paired t-tests of the first and last driven module. Integrated Driving Scores (IDS, Z-scores) will be calculated after logistic regression of driving parameters.

For secondary parameter-based models, we will first use linear mixed models and stepwise testing will be based on biological/plausibility justification rather than purely statistical significance.

Akaike Information Criterion (AIC) and k-fold cross-validation will be used to for prediction models of sleep onset/sleep accidents on the level of individual 40-min-long runs, 4-min-long modules or shorter time units to be explored. For exploring more complex models and potentially prediction models, we will include multiple secondary variables. Rather than on the determination of the averaged latency over 4 runs within a test, such analyzes will be performed per each 40-min-long run (N_run_= 4 x 54 = 216), per 4-min-long module (N_Module_ = 2160) or as running time-series-analysis in shorter time units to be explored, embracing an EPV = 10-15 (observations per predictor variable). Here we plan to test predicting sleep onset and/or sleep accident based on the selected variables within a run. We will employ Akaike Information Criterion (AIC) or AICc for small sample sizes (18): AIC-based model selection helps prevent overfitting by identifying the most parsimonious model rather than selecting significant variables arbitrarily. Cross-Validation techniques (e.g., k-fold cross-validation) will be implemented to ensure robustness and avoid overfitting. Although this approach does not rely on p-values from multiple tests but penalizes model complexity and avoids spurious associations, we still might use False discovery rate (FDR) approaches to help adjusting for false positives while maintaining statistical power. In this context Bonferroni Corrections will be too conservative and could lead to excessive type II errors (false negatives) rather than avoiding and adjusting for type I errors (false positives).

Significant findings from exploratory analyzes will be interpreted cautiously and will most likely require independent validation in future studies.

We cannot reliably aim at uncovering gender differences, but will include sex or gender in any model for control.

The statistical software R, Python, and GraphPad Prism will be used.

Any deviation from the original statistical plan will be described and justified in the final trial report. We intend to complete the study with N_total_ = 54, except recruitment success is (deemed) prohibitive within the projected study duration (LPLV 30/04/2027, see section 4.3) and possible extension of the study duration.

## 5.2 Feature description

*Test-Latency:*

Mean MWT- or DS-MWT-latencies are determined by averaging latencies from four max. 40 min test- sessions determined by AASM-criteria (REF,) and previously reported additional/complementary parameters (e.g., sleep accidents with closed eyes, (1)) for the two tests, respectively.

*Driving Parameters*

We will collect multiple driving parameters, such as the standard deviation of lane position, SDLP, the standard deviation of velocity, SDS, number of lane departures, and LD. Collected parameters will also be used as one criterion to determine the sleep-accident-derived latency for the DS-MWT. IDS

*ET Features*

A recent review showed that eye movement features and the sleep-wake predictor model could be used to predict sleepiness (6). For example, the blink rate increases with increased sleep pressure and can be used to assess drowsiness or sleepiness. Other valuable ET metrics (5) for our setting are the saccade latency, saccade peak velocity and smooth pursuit velocity gain, which could also reflect levels of sleep deprivation and extreme sleepiness.

*EEG Features*

EEG recordings are required to determine MWT-latencies, based on precise identification of sleep onset (SO). According to AASM classification, sleep onset is defined as the start of the first epoch scored as any stage other than stage W. In most individuals this will usually be the first epoch of stage N1, characterized by SEM (slow eye movements) and LAMF (low-amplitude, mixed-frequency) in EEG activity. Electrophysiological data collected during both classical MWT and DS-MWT will provide useful additional insights into the sleep-wake transition. For example, microsleep episodes (MSEs), i.e., brief periods of sleep intrusion during wakefulness, may occur even before sleep onset. We will analyze manually- and automatically-detected MSEs (17).

*PSG Features*

PSG monitors multiple physiological signals, including body position, respiratory movement, electrocardiogram (ECG), EEG, electrooculogram (EOG), electromyogram (EMG), breath airflow and oxygen saturation. PSGs provide a reference or baseline for sleep quality of the night prior to each measurement session. Here, the sleep quality will be measured over several criteria such as duration and occurrence of sleep stages; intensity, continuity and stability of sleep episodes etc. Recording of the breath airflow and oxygen saturation will also allow to derive OSA-associated metrics, such as AHI (apnea-hypopnea index), ODI (oxygen desaturation index) etc.

*Biomarkers (saliva, blood)*

Recent studies in proteomics showed promising findings of sleep-deprivation-associated proteins in human blood serum (9-11). For example, 66 out of 494 proteins were found to be differentially expressed proteins (DEPs) after 6 hours of sleep deprivation in (9). Collecting saliva and blood samples (blood, serum or dried spots) in the MWT and DS-MWT setting will be used for targeted screening for sleep-deprivation-associated proteins.

## 5.3 Handling of missing data and drop-outs

Missing datasets due to participants’ withdrawal from the trial will not be multiply imputed due to the low sample size and inherently low statistical power. All available data will be used, if possible, for statistical analysis. Drop-outs during the study phase will be replaced by recruitment of new participants from the accessible participant pool, if possible.

# Regulatory Aspects AND SAFETY

## 6.1 Local regulations / Declaration of Helsinki

This study is conducted in compliance with the protocol, the current version of the Declaration of Helsinki, the ICH-GCP, the HRA as well as other locally relevant legal and regulatory requirements.

## 6.2 (Serious) Adverse Events and notification of safety and protective measures

An Adverse Event (AE) is any untoward medical occurrence in a patient or a clinical investigation subject which does not necessarily have a causal relationship with the trial procedure. An AE can therefore be any unfavorable or unintended finding, symptom, or disease temporally associated with a trial procedure, whether or not related to it.

A Serious Adverse Event (SAE) (ClinO, Art. 63) is any untoward medical occurrence that

- Results in death or is life-threatening,
- Requires in-patient hospitalization or prolongation of existing hospitalization,
- Results in persistent or significant disability or incapacity, or
- Causes a congenital anomaly or birth defect.

Both Investigator and Sponsor-Investigator make a causality assessment of the event to the trial intervention, (see table below based on the terms given in ICH E2A guidelines). Any event assessed as possibly, probably, or definitely related is classified as related to the trial intervention.

| **Relationship** | **Description** |
| --- | --- |
| Definitely | Temporal relationship  Improvement after dechallenge*  Recurrence after rechallenge  (or other proof of drug cause) |
| Probably | Temporal relationship  Improvement after dechallenge  No other cause evident |
| Possibly | Temporal relationship  Other cause possible |
| Unlikely | Any assessable reaction that does not fulfil the above conditions |
| Not related | Causal relationship can be ruled out |
| *Improvement after dechallenge only taken into consideration, if applicable to reaction | |

Both Investigator and Sponsor-Investigator make a severity assessment of the event as mild, moderate or severe. Mild means the complication is tolerable, moderate means it interferes with daily activities and severe means it renders daily activities impossible. Other grades of severity, such as the terminology from the CTCAE, may be used if appropriately referenced.

**Reporting of SAEs** (see ClinO, Art. 63)

All SAEs are documented and reported immediately (within a maximum of 24 hours) to the Sponsor-Investigator of the study.

If it cannot be excluded that the SAE occurring in Switzerland is attributable to the intervention under investigation, the Investigator reports it to the Ethics Committee via BASEC within 15 days.

Exemptions from expedited reporting may be possible if the SAE is either a clear result of the underlying disease or well-known. Please define those SAEs that are exempted from expedited reporting. A template of the SAE is available at [www.swissethics.ch](http://www.swissethics.ch).

**Follow-up of (Serious) Adverse Events**

Participants who terminate the clinical trial (regularly or prematurely) due to

- reported, ongoing SAE, or
- persistent SAE, e.g. laboratory values or alarming vital signs,

are examined during a follow-up visit. This visit will take place up to 30 days after the end of the study treatment phase. Information from this follow-up visit will be recorded accordingly in the CRF.

[**Notification of safety and protective measures**](https://www.admin.ch/opc/en/classified-compilation/20121176/index.html#a37) (see ClinO, Art 62, b)

If immediate safety and protective measures have to be taken during the conduct of the study, the investigator notifies the Ethics Committee of these measures, and of the circumstances necessitating them, within 7 days.

## 6.3 (Periodic) safety reporting

An annual safety report (ASR) is submitted once a year to the local Ethics Committee by the Investigator (ClinO, Art. 43 Abs 1).

## 6.4 Radiation

n.a., since no interventions with radiation of any kind are planned for this study.

## 6.5 Pregnancy (if applicable)

n.a. since there is no known risk arising from the intervention or study procedure with respect to pregnancy, pregnancy is not an exclusion criterion and will not be handled differently in the analysis.

## 6.6 Amendments

Substantial changes to the study setup and study organization, the protocol and relevant study documents are submitted to the Ethics Committee for approval before implementation. Under emergency circumstances, deviations from the protocol to protect the rights, safety and well-being of human subjects may proceed without prior approval of the Ethics Committee. Such deviations shall be documented and reported to the Ethics Committee as soon as possible.

Substantial amendments are changes that affect the safety, health, rights and obligations of participants, changes in the protocol that affect study objective(s) or central research topic, changes of study site(s) or of study leader and sponsor (ClinO, Art. 29).

A list of substantial changes is also available on [www.swissethics.ch](http://www.swissethics.ch).

A list of all non-substantial amendments will be submitted once a year to the competent EC together with the ASR.

## 6.7 Notification and reporting upon completion, discontinuation or interruption of the study

Upon regular study completion, the Ethics Committee is notified via BASEC within 90 days (ClinO, Art. 38).

The Sponsor-Investigator may terminate the study prematurely according to certain circumstances, e.g.

- Ethical concerns,
- Insufficient participant recruitment,
- When the safety of the participants is doubtful or at risk (e.g. when the benefit-risk assessment is no longer positive),
- Alterations in accepted clinical practice that make the continuation of the study unwise, or
- Early evidence of harm or benefit of the experimental intervention

Upon premature study termination or study interruption, the Ethics Committee is notified via BASEC within 15 days (ClinO, Art. 38).

A final report is submitted to the Ethics Committee via BASEC within a year after completion or discontinuation of the study unless a longer period is specified in the protocol (ClinO, Art. 38).

## 6.8 Insurance

The insurance is covered by the "Insurance for Clinical Trials and Non-Clinical Trials" of the Zurich Insurance Company. This covers all damages associated with the clinical trial. In the event of study-related damage or injuries, the liability of this institution provides compensation, except for claims that arise from misconduct or gross negligence.

Participants must strictly follow the instructions of the study team in order not to jeopardize insurance coverage. Furthermore, they may not undergo any other medical treatment during the clinical trial without the consent of the investigator (except in emergencies). They must inform the investigator immediately of any emergency treatment. In the event of health problems or other damage during or after participation in the study, the subject must be informed. In the event of claim, representatives of the insurance company are also given access to the medical data via the examiner, but only to the extent necessary to settle the claim. A copy of the insurance certificate is stored in the test center folder.

# 7 FURTHER Aspects

## 7.1 Overall ethical considerations

Study procedure and intervention are associated with minimal risks and burdens because the procedures are mainly standardized and participants are already used to conduct drug holidays. The only side-effect of the study procedure might be symptoms linked to kinetosis during the driving simulation but these will rapidly subside. The study design in section 3.3 is appropriate for the study population. The study team is adequate for this small exploratory study. Hence, ethical considerations with respect to risks are minimal.

We will not include any vulnerable participants.

The study design described in section 3.3 is appropriate for the study population. The study team is adequate for this small exploratory study.

Due to the exploratory nature of the study and an expected gender imbalance (because of higher prevalence in males), the results might not yet be generalizable.

The study addresses a clear scientific gap and opens up a new, relatable and understandable measurement of excessive daytime sleepiness (EDS). This way, it addresses and improves safe driving. Thus, this and similar subsequent studies will potentially be highly informative for the OSA-affected drivers with additional potential implications for traffic medicine assessments, policy making and overall understanding of the risks associated with CPAP-holidays (i.e. <7-day-long voluntary CPAP withdrawal).

## 7.2 Risk-benefit assessment

The participation in this study involves only minimal risks (category A). The study procedures at each one of 7 visits consist of standardized and mostly non-invasive tests. Participants are under constant supervision.

In rare cases, the use of the driving simulator can lead to dizziness and discomfort (simulator sickness), but these quickly subside when leaving the simulator or completing the test.

EEG-mounting and blood sampling by means of peripheral venous donor catheter will be conducted by trained personnel. Hematoma from blood sampling and/or skin irritations from EEG electrodes may occur rarely, but these usually disappear without further complications. The collected full blood volume of 9ml ml is generally considered harmless to health. There are no health risks in the sampling of urine, saliva, dried blood spots, and exhaled breath.

For the intervention “CPAP-withdrawal” (W), participants omit their overnight use of CPAP-treatment at home for a ≥7-day-period (typically holidays) and thus will have a reactivation of OSA. This intervention has been validated as a safe, effective and well-controlled model to study the pathophysiological consequences of OSA (3, 4). All patients will be primarily assessed by physicians from the Sleep Disorders Center on whether a CPAP-withdrawal can cause them health risks. Only preselected patients with no risks to their health will be included in the study. At the end of the study, patients will resume their established long-term CPAP therapy. Professional drivers will be excluded from the study, if working during the study period.

Otherwise, participation in the study is not associated with any known specific risks.

There is no direct health benefit for the study participants.

Overall social and scientific benefits are described in section 7.1.

# 8 Quality CONTROL AND Data protection

## 8.1 Quality measures

For quality assurance the sponsor, the Ethics Committee or an independent trial monitor may visit the research sites. Direct access to the source data and all study-related files is granted on such occasions. All involved parties keep the participant data strictly confidential.

All study team members are trained in accordance with their assigned roles. Staff is trained to ensure reproducible and high data and analysis quality. The coordinating center of the monocentric study is based at the Division of Traffic Medicine. There are no steering and no endpoint adjudication committees appointed in this exploratory study. The data management team is identical to the investigators and part of the Division of Traffic Medicine.

## 8.2 Data recording and source data

For each trial participant, the investigators complete a paper Case Report Form (CRF), in which the acquisition of all trial-relevant tests of a participant are recorded.

The names of included participants and their corresponding participant numbers (PID, attributed strictly according to sequence of inclusion) are recorded in a separate subject identification log. This log will also contain exclusion (i.e. after screening or non-adherence) or voluntary drop-out by date.

All data fields of the CRF must always be filled out legibly and clearly with a black or blue ballpoint pen to ensure that all copies are clearly legible. Corrections should be made so that the old entry remains readable (the use of correction fluid is not allowed). Corrections must be entered, signed, and dated by the examiner. Data that is not available or was not collected must be clearly marked as such (n.a.). The investigators certify with their signature that the entries in the CRF are correct.

CRFs must be kept up to date to reflect the status of a participant in each study phase. The name of the trial participant must not be visible in the CRF. The patients’ information on the individual CRFs is coded by means of a participant number (PID) to protect personal data. Initials must not be used together with the date of birth in the CRF. Furthermore, it must be ensured that every person who can make entries and corrections in the CRF can be identified. A list with the signature and abbreviation of all authorized persons is kept in the investigator site file. The documented medical history and written statements on the status of the participating person during the clinical trial are to be kept together with the CRF of the respective participating person. These records may also include the following: original or copies of laboratory results, other medical or psychological tests, questionnaires, etc. The investigators ensure complete and accurate documentation of participant data in the CRF. All data entered into the CRF must also be found in the patient file, either as printouts or as notes by the examiner or another person delegated by the examiner. Exceptions are data for which the entries in the CRF are considered source data. These must be precisely defined in advance for each trial.

All essential documents of the clinical trial must be kept for at least 20 years after the end or termination of the clinical trial. Study records and other original data must be kept for the longest possible period permitted by the Institute for Forensic Medicine.

The following documents are considered source data:

- AE and SAE forms
- Notes from study personnel
- Medical records from other departments or hospitals, or discharge reports (findings, medical letters) or correspondence with other departments/hospitals, if the trial participant was treated there during the clinical trial or during the follow-up phase.

The source data must be available at the trial site to verify the existence of the trial participants and to confirm the accuracy and completeness of the data. Source data must contain both trial-relevant original documents and the medical history and treatment of the trial participants. The following information must be included at a minimum in the source data:

- Demographic data (age, sex)
- Details of inclusion and exclusion criteria
- Dated and signed informed consent forms of the trial participants
- Visit dates
- Information on the sequence of interventions
- Details of medical history and physical examinations
- SAEs and concomitant medication
- Results of relevant examinations
- Laboratory printouts
- Details of the delivery and return of trial materials related to the intervention
- Reasons for early withdrawal

The following information is used directly from the source data for analysis and is not transferred to the CRF.

- Measurement protocols
- Questionnaires (e.g., ESS, KSS)
- Data recordings (SILAB, SAFE, eye tracking, EEG)
- Laboratory results (blood level, drug test, exhalomics data)

The storage location of source data (raw data) is noted in the CRF, see CRF section 8. Appendix – Data Storage Log.

## 8.3 Confidentiality and coding

Trial and participant data will be handled with uttermost discretion and is only accessible to authorized personnel who require the data to fulfil their duties within the scope of the study. On the CRFs and other study-specific documents, participants are only identified by a unique participant number.

Investigators are responsible for ensuring that all study-related information and data collected are kept strictly confidential. Any disclosure of information to people who are not directly involved in the study must be approved by the owner of the data. Data collection, disclosure, archiving and analysis of personal data within the study are carried out strictly in accordance with the applicable Swiss data protection regulations. The prerequisite for this is the voluntary consent of the trial participants, which is given by signing the declaration of consent before participating in the clinical trial. The data collected as part of this clinical trial can be viewed for verification or inspection by monitors and the independent Ethics Committee.

No genetic data will be collected in this exploratory study, thus not encoded.

Biological material in this study is not identified by participant name but by a unique participant number, PID. Biological material is transferred to the restricted analyzing laboratory only in person by the authorized study personnel. Biological material is appropriately stored and analyzed in a restricted area only accessible to the authorized personnel. Resulting biological data is stored and analyzed in a restricted area, only accessible to authorized personnel.

## 8.4 Retention and destruction of study data and biological material

All study data are archived for 20 years at the Institute for Forensic Medicine of the University of Zurich after study termination or premature termination of the study. It is not planned to establish a biobank.

# 9 MonitoriNg and Registration

This study is another Clinical Trial according to ClinO, Chapter 4 with minimal risks and burdens for the already well-experienced OSA participants in conducting CPAP-withdrawal. According to ClinO Art. 3 Abs. 3, a risk-adapted monitoring will be ensured.

Internal monitoring duties will be carried out by Dr. med. Kristina Keller, who is not actively taking part in recruitment, data acquisition or data analysis. There is no additional external monitoring foreseen or required.

All source data/documents are accessible to the internal monitors. Questions are answered during monitoring, where available documentations will be evaluated.

Internal monitoring visits will be carried out unannounced by the internal monitor. There are no steering and no endpoint adjudication committees appointed in this exploratory study. The data management team is part of the Division of Traffic Medicine. The coordinating center is based at the Division of Traffic Medicine. There is no need for a data management committee. Data analysis will take place after trial completion and independent of the (partial) funding (see section 10).

This study will be registered at clinicaltrials.gov as well as in the Swiss National Clinical trial Portal (SNCTP via BASEC).

# 10. Funding / Publication / declaration of Interest

This study is supported (partly funded) by the Emma-Louise-Kessler-Fund (ELK).

The results of this study will be published in peer-reviewed scientific journals, regardless of the direction of outcomes. Digital copies will be sent to trial participants if they express their interest. Findings will also be presented at scientific conferences and meetings. A final project report will be sent to the funder.

The authors declare no financial or other competing interests.

# 11. REFERENCES

1. Pisteljic M, Keller K, Lakamper S. Capturing sleep accidents in driving simulation as a promising tool to assess excessive daytime sleepiness with high ecological validity-a pilot study. Sleep. 2024;47(8).

2. McCall CA. Moving towards a more naturalistic approach to evaluating drowsy driving risk. Sleep. 2024;47(8).

3. Schwarz EI, Martinez-Lozano Sinues P, Bregy L, Gaisl T, Garcia Gomez D, Gaugg MT, et al. Effects of CPAP therapy withdrawal on exhaled breath pattern in obstructive sleep apnoea. Thorax. 2016;71(2):110-7.

4. Schwarz EI, Stradling JR, Kohler M. Physiological consequences of CPAP therapy withdrawal in patients with obstructive sleep apnoea—an opportunity for an efficient experimental model. Journal of Thoracic Disease. 2018:S24-S32.

5. Aitken B, Downey LA, Rose S, Arkell TR, Shiferaw B, Hayley AC. Driving performance and ocular activity following acute administration of 10 mg methylphenidate: A randomised, double-blind, placebo-controlled study. J Psychopharmacol. 2024;38(11):998-1006.

6. Roy D, Nah FF-H, editors. A Review on Eye-Tracking Metrics for Sleepiness. HCI International 2020–Late Breaking Papers: Interaction, Knowledge and Social Media: 22nd HCI International Conference, HCII 2020, Copenhagen, Denmark, July 19–24, 2020, Proceedings 22; 2020: Springer.

7. Skorucak J, Hertig-Godeschalk A, Achermann P, Mathis J, Schreier DR. Automatically Detected Microsleep Episodes in the Fitness-to-Drive Assessment. Front Neurosci. 2020;14:8.

8. Roeder M, Bradicich M, Schwarz EI, Thiel S, Gaisl T, Held U, et al. Night-to-night variability of respiratory events in obstructive sleep apnoea: a systematic review and meta-analysis. Thorax. 2020;75(12):1095-102.

9. Bjorkum AA, Griebel L, Birkeland E. Human serum proteomics reveals a molecular signature after one night of sleep deprivation. Sleep Adv. 2024;5(1):zpae042.

10. Geyer PE, Voytik E, Treit PV, Doll S, Kleinhempel A, Niu L, et al. Plasma Proteome Profiling to detect and avoid sample-related biases in biomarker studies. EMBO Mol Med. 2019;11(11):e10427.

11. Ignjatovic V, Geyer PE, Palaniappan KK, Chaaban JE, Omenn GS, Baker MS, et al. Mass Spectrometry-Based Plasma Proteomics: Considerations from Sample Collection to Achieving Translational Data. J Proteome Res. 2019;18(12):4085-97.

12. Scholz M, Lakaemper S, Keller K, Dobay A, Steuer AE, Landolt HP, et al. Metabolomics-based Sleepiness Markers for Risk Prevention and Traffic Safety (ME-SMART): a monocentric, controlled, randomized, crossover trial. Trials. 2023;24(1):131.

13. Cohen J. Statistical power analysis for the behavioral sciences. 2nd ed ed. Hillsdale, NJ: Lawrence Erlbaum Associates; 1988 1988.

14. Moore DS, McCabe GP, Craig BA. Introduction to the practice of statistics. Ninth edition ed. New York: W.H. Freeman, Macmillan Learning; 2017.

15. Bonnet MH, Arand DL. Impact of motivation on multiple sleep latency test and maintenance of wakefulness test measurements. Journal of Clinical Sleep Medicine. 2005;1(04):386-90.

16. Banks S, Barnes M, Tarquinio N, Pierce RJ, Lack LC, Doug McEvoy R. Factors associated with maintenance of wakefulness test mean sleep latency in patients with mild to moderate obstructive sleep apnoea and normal subjects. Journal of sleep research. 2004;13(1):71-8.

17. Chow S-C, Liu J-P. Design and analysis of bioavailability and bioequivalence studies: CRC press; 1999.

18. Isler Y, Schwab S, Wick R, Lakamper S. Strong evidence for age as the single most dominant predictor of medically supervised driving test-mini mental status test outcomes provide only weak but significant moderate additional predictive value. BMC Geriatr. 2022;22(1):247.

Appendix 1: Schedule of assessments (if applicable)

See Tables 1 and 2 in section 4.3.
