# Supplementary material for: Validating a novel driving simulation-based MWT against the standard MWT in an OSA-cohort challenged by CPAP-withdrawal (DS-MWT2) – Protocol for a monocentric, controlled, randomized, crossover trial
Source: PLoS One. 2026 Jul 9;21(7):e0341997. doi: 10.1371/journal.pone.0341997 (PMC13349176; doi:10.1371/journal.pone.0341997)
Supplement: Attachment — Submitted filename: Response to Reviewers.docx [file pone.0341997.s003.docx]

Comments and Responses are numbered in sequence:

Editor-in chief comments:

**Comment 1:**
Please ensure that your manuscript meets PLOS ONE's style requirements, including those for file naming. The PLOS ONE style templates can be found at [links provided].

**Response:**
Thank you very much for this helpful comment. We have carefully checked the manuscript against the PLOS ONE style requirements and formatting templates. The section names and the order of sections have been revised accordingly to comply with the journal’s required structure. In addition, all files have been renamed and re-uploaded using the correct file naming format.

**Comment 2:**
Please note that PLOS One has specific guidelines on code sharing for submissions in which author-generated code underpins the findings in the manuscript. In these cases, we expect all author-generated code to be made available without restrictions upon publication of the work. Please review our guidelines at the provided link and ensure that your code is shared in a way that follows best practice and facilitates reproducibility and reuse.

**Response:**
No author-generated code was used in this study protocol manuscript, as the manuscript describes the study design and methodology and does not report study results or analyses. Nevertheless, a statement is included in the “Data Availability” section specifying that all code used for future statistical analyses within the study will be made publicly available upon publication of the study results.

**Comment 3:**
Thank you for stating the following in the Acknowledgments Section of your manuscript: [...] Please remove any funding-related text from the manuscript and let us know how you would like to update your Funding Statement. [...]

**Response:**
All funding-related information was removed from the manuscript text and acknowledgments section in accordance with the journal requirements. The funding statement was revised accordingly, and the updated funding information was included in the revised cover letter submitted together with this revision.

**Comment 4:**
We note that the grant information you provided in the ‘Funding Information’ and ‘Financial Disclosure’ sections do not match. When you resubmit, please ensure that you provide the correct grant numbers for the awards you received for your study in the ‘Funding Information’ section.

**Response:**
The funding information and financial disclosure statements were carefully revised and harmonized to ensure consistency across all submission documents. The corrected funding statement and grant information were included in the revised cover letter submitted together with this document.

**Comment 5:**
Thank you for stating the following financial disclosure: [...] Please state what role the funders took in the study. If the funders had no role, please state: “The funders had no role in study design, data collection and analysis, decision to publish, or preparation of the manuscript.”

**Response:**
The funding statement was revised using the requested wording to clarify the role of the funders. The updated statement was included in the revised cover letter submitted together with this document.

**Comment 6:**
Your ethics statement should only appear in the Methods section of your manuscript. If your ethics statement is written in any section besides the Methods, please delete it from any other section.

**Response:**
The ethics statement was moved to the Methods section of the manuscript and removed from all other sections accordingly.

**Comment 7:**
We note that the original protocol that you have uploaded as a Supporting Information file contains an institutional logo. As this logo is likely copyrighted, we ask that you please remove it from this file and upload an updated version upon resubmission.

**Response:**
All institutional logos were removed from the protocol file, and the updated document was re-uploaded with the revised submission.

**Comment 8:**
If the reviewer comments include a recommendation to cite specific previously published works, please review and evaluate these publications to determine whether they are relevant and should be cited. There is no requirement to cite these works unless the editor has indicated otherwise.

**Response:**
No specific recommendations for additional citations or previously published works were provided by the reviewers.

Reviewer 1 comments:

This is a very ambitious study with one primary outcome and several secondary outcomes comparing MWT sleep latencies with DS-MWT latencies unti sleep accidents.
Some comments:
The study goal is a good one. One of the major aims to develop a cost saving method is certainlay unrealistic. The MWT results are strongly motivation dependent and dependent on the lack of distraction for the probands. As such the DS-MWT is a contradiction to MWT protocol. Despite the extremely detailed protocol this remains the major flaw.
line 233: Why perform home PSG several times? Is it to confirm sleep duration of 7 h or to confirm sleep quality? If it is for sleep duration actimetry would do as well and even assess sleep duration during the run-in time and througout all nights. Actimetry would also save costs.
Line 240: what is the sitting position in the non DS-MWT? Semirecumbent or sitting in a kind of car seat?
Line 278: it is difficult to understand how different latency definitions (MWT, vs DS-MWT) can be compared. It can be expected that those who stay on CPAP will not have necessarily have an „eyes closed“ event.
Line 298: Which EEG-based markers of EDS are exactly meant?


MWT results are highly dependent on motivation. Filling out all the tests before and after MWT, the montage, the verbal interventions and the known study goal, motivation could be strongly biased.
Line 347: What does SA stand for?
Lin 366: Are there any stops or cars passing by included in the driving simulation (trucks, traffic lights, speed changes…?)

**Response:**

We thank the reviewer for the careful evaluation of our manuscript and for the constructive comments and suggestions. The point-by-point responses follow below.

**Point 1:**
The study goal is a good one. One of the major aims to develop a cost saving method is certainlay unrealistic.

**Response:**
The development of a cost-saving method is not an intended aim of the study. We believe this impression may have resulted from the statement in lines 33–34: “Moreover, the cost and time demands of MWTs limit their feasibility for widespread application in driving license certification.” This sentence was intended to provide context regarding practical limitations of the current MWT procedure rather than to define a study objective. The primary aim of the study is the validation of an additional ecologically relevant assessment tool in the context of fitness-to-drive evaluation, rather than replacement of the classical MWT or resolution of all its practical limitations. The manuscript was revised to clarify this point in lines: 39-41.

**Point 2:**
The MWT results are strongly motivation dependent …

**Response:**
We agree that motivation represents an important factor influencing MWT performance and appreciate the reviewer for highlighting this aspect. In fact, this limitation constitutes one of the motivations for the development of additional ecologically valid approaches for fitness-to-drive (FTD) assessment. As stated in lines 31–33 of the manuscript, previous literature has discussed the potential influence of individual motivational factors on MWT performance in the context of FTD evaluation, for example in situations involving potential loss of a driving license or other personal incentives affecting test performance. Importantly, within the present study design, participants do not experience any direct legal consequences based on their test performance, and therefore substantial motivation-related bias is not expected. In addition, the randomized crossover design minimizes the influence of stable interindividual motivational differences, assuming no major changes in participant motivation over the relatively short study period.

The influence of motivation on performance in ecologically valid driving simulation-based wakefulness assessments remains an important topic and may be addressed in future dedicated studies.

**Point 3:**
The MWT results are dependent on the lack of distraction for the probands. As such the DS-MWT is a contradiction to MWT protocol. Despite the extremely detailed protocol this remains the major flaw.

**Response:**
We agree that the classical MWT intentionally minimizes external stimulation in order to create soporific conditions. However, this characteristic also represents one of the key limitations that the present study aims to address in the context of FTD assessment. Real-world driving inherently involves continuous sensory input, environmental interaction, and sustained task engagement. Therefore, the DS-MWT is not intended to replicate the classical MWT conditions directly, but rather to complement them by providing a more ecologically valid context in which wakefulness and driving-related performance can be assessed.

At the same time, it cannot be assumed a priori that DS-MWT latencies will necessarily be longer than classical MWT latencies. The continuous cognitive demand required to maintain lane position and vehicle distance may itself contribute to earlier performance deterioration and earlier test termination (sleep accident). This relationship remains to be systematically evaluated within the present study.

**Point 4:**
Line 233: Why perform home PSG several times? Is it to confirm sleep duration of 7 h or to confirm sleep quality? If it is for sleep duration actimetry would do as well and even assess sleep duration during the run-in time and throughout all nights. Actimetry would also save costs.

**Response:**
Repeated home PSG recordings are performed primarily to assess sleep quality and OSA-related parameters, including sleep architecture, arousals, sleep efficiency, and respiratory events, which may strongly influence next-day wakefulness and driving-related outcomes (lines 384–393). In addition, repeated PSG assessments allow control for night-to-night variability and potential order effects within the crossover study design.

While actimetry may represent a useful complementary measure for estimating sleep duration and sleep-wake patterns, it is not sufficient as a standalone method in an OSA population, as it cannot reliably capture sleep architecture or breathing disturbances. Furthermore, the repeated PSG recordings support the exploratory aims of the study investigating associations between nocturnal physiological parameters and next-day performance measures.

**Point 5:**
Line 240: What is the sitting position in the non DS-MWT? Semirecumbent or sitting in a kind of car seat?

**Response:**
Participants in the standard MWT are positioned in a comfortable semi-recumbent supported sitting position in accordance with AASM (2021) guidelines. This setup is broadly comparable to a typical driving posture, including back and head support. We thank the reviewer for pointing out this important methodological detail relevant for study replication. The corresponding clarification was added to the manuscript (lines 308–311).

**Point 6:**
Line 278: It is difficult to understand how different latency definitions (MWT vs DS-MWT) can be compared. It can be expected that those who stay on CPAP will not necessarily have an “eyes closed” event.

**Response:**
The manuscript was revised to clarify the latency definitions and their comparison (currently lines 351-354). In addition, the introduction (lines 46-48) more explicitly emphasizes that, compared with the solely EEG-based criteria used in the classical MWT, the DS-MWT latency definition is complemented by an additional “naturalistic” criterion: an accident occurring with closed eyes (“sleep accident”, SA).

Regarding the reviewer’s concern, current consensus definitions of sleep and sleep onset according to AASM criteria inherently require eye closure as part of the physiological sleep process. However, it possible that participants continuing CPAP treatment may not experience an “eyes closed” event during either condition. However, this outcome would be consistent with the expectation of preserved wakefulness and reduced sleepiness under effective treatment. Similarly, participants in both the classical MWT and DS-MWT may complete trials without reaching sleep-related endpoints.

**Point 7:**
Line 298: Which EEG-based markers of EDS are exactly meant?

**Response:**
We thank the reviewer for pointing out that the description of EEG-based markers of EDS was incomplete. The corresponding section was carefully revised and expanded to specify the EEG-based markers considered in the study. Additional literature was also cited to support and clarify this aspect (lines 375–381).

**Point 8:**
MWT results are highly dependent on motivation. Filling out all the tests before and after MWT, the montage, the verbal interventions and the known study goal, motivation could be strongly biased.

**Response:**
The EEG montage is performed on the day prior to the experimental session and therefore does not directly interfere with the testing procedure itself. In addition, demographic and baseline questionnaires are completed during the screening visit rather than immediately before testing. Only the Karolinska Sleepiness Scale (KSS) is administered directly before each test session.

Furthermore, all verbal interventions and procedural interactions are highly standardized and are performed identically for both the MWT and DS-MWT conditions. The study information provided to participants describes the project only as a comparison of two wakefulness assessment methods and does not imply any personal consequences or benefits related to fitness-to-drive evaluation or individual test performance. Therefore, substantial systematic motivational bias is not expected.

**Point 9:**
Line 347: What does SA stand for?

**Response:**
SA stands for “sleep accident.” The term was previously defined in the manuscript (line 48) and is also included in the abbreviation list at the end of the manuscript (page 18).

**Point 10:**
Line 366: Are there any stops or cars passing by included in the driving simulation (trucks, traffic lights, speed changes…?)

**Response:**
We thank the reviewer for pointing out the incompleteness of the driving scenario description. The corresponding section was revised and expanded to provide a more detailed description of the simulator environment and traffic conditions, including the characteristics of surrounding traffic and driving events (lines 483–490).

Reviewer 2 comments:

The investigators have already piloted a Driving Simulation–based MWT (DS-MWT), designed to evaluate sleepiness in an ecologically valid driving scenario. The present study aims to validate the novel DSMWT against the classical MWT in a functionally relevant cohort of patients with obstructive sleep apnea (OSA). The primary outcome measure of this trial will be the difference in mean latency between the classical MWT and the DS-MWT. The secondary hypotheses (objectives) are noted in the synopsis of the supplemental protocol and elsewhere. The elements of the planned protocol are in place. Further clarification is needed as will be noted below. My focus is on the statistical design and analysis of the project. The schematic outline in the protocol plan looks reasonable as do other elements. Also, see Figures 1 and 2 in the manuscript and Table 1 and 2 in the supplementary protocol.

1. Define clearly in this context the terms ‘latency’ and ‘causality assessments’. Be careful of the term ‘causality’. The more appropriate term may be ‘association’ in this statistical context. Defend the use of, ‘causality’.

2. The analysis section in the protocol lacks detail. Be more specific in section 5.1.2 stating the exact analysis for targeted objectives (with dependent variables and predictors including possible interactions).

3. In the section 5.1.2 what exactly is ‘logistic regression of driving parameters’? Please expand.

4. Explain the rationale for the 2 to 1 intervention to control imbalance.

5. In the sample size section, 5.1.1 of the protocol and text, please give the numerical value of the ICC and how was it derived?

6. The data management section is not complete. What are the data quality control or validation procedures even though they may be referenced?

Please do a thorough edit of the manuscript to be sure that other detail is included. The document should be self contained.

**Response**: We thank the reviewer for the careful evaluation of our manuscript and for the constructive comments and suggestions. The point-by-point responses follow below.

**Point 1:**
Define clearly in this context the terms ‘latency’ and ‘causality assessments’. Be careful of the term ‘causality’. The more appropriate term may be ‘association’ in this statistical context. Defend the use of ‘causality’.

**Response:**
The latency definitions are described in the manuscript for both tests. The classical MWT latency is defined in lines 27–30, while the DS-MWT latency definition is provided in lines 46–47. Further elaboration regarding the definitions and comparison of both latency measures is provided in lines 351–357.

Regarding the reviewer’s concern about “causality,” we would like to clarify that the term “causality” was not used in the manuscript. Associations and relationships between variables are described using appropriate non-causal terminology.

**Point 2:**
The analysis section in the protocol lacks detail. Be more specific in section 5.1.2 stating the exact analysis for targeted objectives (with dependent variables and predictors including possible interactions).

**Response:**
We appreciate the reviewer’s comment and revised the statistical analysis section for the primary outcome accordingly (lines 592–604). The revised section now provides a more detailed description of the primary hypothesis testing strategy and the corresponding statistical approach. Secondary outcomes remain exploratory in nature; therefore, not all potential analyses and interactions were predefined in detail within the protocol.

**Point 3:**
In section 5.1.2 what exactly is “logistic regression of driving parameters”? Please expand.

**Response:**
As these analyses belong to the exploratory secondary outcomes, the protocol intentionally allows flexibility regarding the exact statistical approaches used for future exploration of multimodal driving-related parameters. However, we agree that the original wording was insufficiently specified and could be confusing for the reader. Therefore, the statement regarding “logistic regression of driving parameters” was removed from the manuscript.

**Point 4:**
Explain the rationale for the 2 to 1 intervention to control imbalance.

**Response:**
There is no intended 2:1 allocation ratio in the study design. The apparent imbalance results from two independent sample size calculations performed for different study populations and objectives.

**Point 5:**
In the sample size section, 5.1.1 of the protocol and text, please give the numerical value of the ICC and how was it derived?

**Response:**
We thank the reviewer for pointing out the absence of the numerical ICC value. The corresponding information was added to the manuscript. Because no directly applicable ICC for repeated MWT measurements under the present experimental conditions was available, we prespecified a moderate within-subject correlation of ρ = 0.5 for planning purposes. This value was chosen as a conservative compromise, as higher ICC values would reduce the required sample size whereas lower ICC values would increase it. To address this uncertainty, the protocol additionally includes an interim assessment to re-evaluate the observed within-subject correlation and the resulting sample size requirements.

**Point 6:**
The data management section is not complete. What are the data quality control or validation procedures even though they may be referenced?

**Response:**
We appreciate the reviewer’s comment and expanded the data management section to provide additional details regarding data quality control and validation procedures (lines 430–433).

Reviewer 3 comments:

The authors present a protocol for a study which is, according to the title of the submitted paper, intended to validate a novel version of the MWT against the standard MWT. The primary objective named by the authors is "to determine whether there is a statistically significant difference in latency between the classical MWT and the DS-MWT". This is unclear. If there is a significant difference, is the new version with the driving simulation not valid? If there is no difference, why should anyone use the new version, which needs much more effort and instrumentation? As a rationale for their modification of the MWT with the inclusion of a driving simulator they argue that the standard MWT “may not accurately reflect real-world driving risks”, and their variation of the MWT might introduce “a realistic, supposedly more ecologically valid alternative to standard sleepiness assessment tools” and “may enhance clinical decision-making regarding fitness to drive (FTD)”. However, the standard MWT has never been adequately validated as a tool to assess FTD, and a critical MWT score which indicates “real-world driving risks” is not established. A validation with respect to FTD or similar safety aspects would need to involve an established gold standard, which is obviously hard to come by. A surrogate parameter, which might provide face validity, but not truly established ecological validity, might be the number of “sleep accidents” as defined by the authors, which, however, seem to occur with this MWT variant in much higher numbers than would be expected in real life (see citation 5). In summary, it is not warranted to call this study a “validation”

Further Issues

The method to induce sleepiness applied in the study is not the same as the one used in the cited papers (cf. cit. 23), and the mentioned paper names a number of factors which influence the degree of change in subjective (not objective) sleepiness in their population. This should be noted, as it may influence the necessary sample size.

As the authors state, "it remains unclear whether EDS itself is consistently and measurably reactivated in this model, as no systematic data on its recurrence are currently available". Therefore, at the very least, the effect of CPAP withdrawal on subjective sleepiness in the patient population should be controlled (e.g., by appropriately modified ESS).

The protocol lacks the assessment of basic vital signs (e.g., weight, height, blood pressure, heart rate) at trial start and after intervention, the latter for safety reasons and to control for effects on the bio-samples.

It is not specified how "sleep quality" and its night-to-night variability are to be assessed in the PSG recordings.

The protocol names some inclusion and exclusion criteria. However, in the following text, it is mentioned that the participants in the patients are referred from a partnering institution, and it is not clear how the referred patients are pre-selected by that institution. The control group, which is relatively small, and the lack of any matching criteria reduces comparability with the clinical sample, even with statistical adaptation methods.

The use of medications with potential influence on sleepiness, and wake promoting agents (such as caffeine or smoking) in the participants is apparently not controlled or the control of these factors is not mentioned.

The large number of secondary outcomes also constitutes an issue. In the paper, many of these outcomes are listed in a rather vague language, and no rationale is given why just these measures are selected, and how they are related to the basic question of the study, i.e., assessment of FTD.

A procedure to make original data available (with proper respect to the protection of patient identity) should be included in the paper.

**Response**: We thank the reviewer for the careful evaluation of our manuscript and for the insightful comments and questions. All the comments are addressed point-by-point below.

**Point 1:**
The primary objective named by the authors is "to determine whether there is a statistically significant difference in latency between the classical MWT and the DS-MWT". This is unclear. If there is a significant difference, is the new version with the driving simulation not valid? If there is no difference, why should anyone use the new version, which needs much more effort and instrumentation?

**Response:**
The primary aim of the study is the validation of an additional ecologically relevant assessment tool in the context of fitness-to-drive evaluation, rather than replacement of the classical MWT or resolution of all its practical limitations. To emphasize this aspect more clearly, the manuscript was revised accordingly (lines 39–41).

A statistically significant difference between MWT and DS-MWT latencies would not necessarily invalidate the DS-MWT approach. Depending on the direction and magnitude of the observed differences, such findings may instead indicate that the two methods capture different aspects of daytime sleepiness and wakefulness relevant to fitness-to-drive assessment, potentially highlighting limitations of the classical MWT in ecologically valid driving contexts. Conversely, the absence of significant differences would suggest that both methods assess similar aspects of daytime sleepiness.

The relevance of the DS-MWT approach lies primarily in its increased ecological validity and higher acceptance among affected populations (as reported in the pilot study). Furthermore, the classical MWT requires highly trained personnel and extensive post hoc EEG scoring to identify sleep-related events. In contrast, simulator-based outcomes such as “sleep accidents” (e.g., a collision occurring with closed eyes during the driving scenario) are automatically registered in real time and may therefore facilitate outcome assessment without requiring extensive post hoc physiological scoring.

**Point 2:**
As a rationale for their modification of the MWT with the inclusion of a driving simulator they argue that the standard MWT “may not accurately reflect real-world driving risks”, and their variation of the MWT might introduce “a realistic, supposedly more ecologically valid alternative to standard sleepiness assessment tools” and “may enhance clinical decision-making regarding fitness to drive (FTD)”. However, the standard MWT has never been adequately validated as a tool to assess FTD, and a critical MWT score which indicates “real-world driving risks” is not established.

**Response:**
We agree with the reviewer that the classical MWT has not been adequately validated as a dedicated tool for FTD assessment and that no universally accepted cut-off defining real-world driving risk currently exists. Indeed, this limitation represents one of the central motivations for the development and investigation of additional approaches specifically designed for the FTD context. Several previous publications have discussed the limitations of current sleepiness assessment methods in relation to driving performance and highlighted the need for more ecologically relevant assessment paradigms (added citations 15, 20).

**Point 3:**
A validation with respect to FTD or similar safety aspects would need to involve an established gold standard, which is obviously hard to come by.

**Response:**
We agree that a true gold standard for objective validation of driving-related sleepiness risk is currently lacking. Direct real-world validation is difficult due to ethical, practical, and safety limitations. Therefore, the DS-MWT approach aims to provide the closest possible approximation to real-world driving conditions within a controlled and standardized laboratory environment.

**Point 4:**
A surrogate parameter, which might provide face validity, but not truly established ecological validity, might be the number of “sleep accidents” as defined by the authors, which, however, seem to occur with this MWT variant in much higher numbers than would be expected in real life (see citation 5).

**Response:**
We would like to clarify that the pilot study (citation 5) did not aim to reproduce real-world accident frequencies or establish predictive driving-risk thresholds, it primarily reports results and shows the feasibility of such approach. Furthermore, the occurrence of a “sleep accident” (SA) itself is not interpreted as evidence that a participant is unfit to drive. Rather, the latency until such an event occurs represents the parameter of interest within the DS-MWT paradigm.

**Point 5:**
In summary, it is not warranted to call this study a “validation”.

**Response:**
We appreciate this important conceptual point. In the context of the present study, the term “validation” refers to the systematic comparison of DS-MWT-derived latency measures with the established classical MWT paradigm, rather than validation against a definitive real-world gold standard for FTD. The study does not aim to establish normative cut-off values or definitive driving-risk thresholds. Such standardization and predictive validation would require future dedicated studies.

**Point 6:**
The method to induce sleepiness applied in the study is not the same as the one used in the cited papers (cf. cit. 23), and the mentioned paper names a number of factors which influence the degree of change in subjective (not objective) sleepiness in their population. This should be noted, as it may influence the necessary sample size.

**Response:**
We would like to clarify that the CPAP withdrawal model used in the present study is supported by the corresponding CPAP withdrawal literature, in particular citation 21 (AJRCCM), where this model was shown to affect both subjective sleepiness and objective vigilance after one and two weeks of CPAP withdrawal.

We agree that patients with OSA represent a clinically heterogeneous population with varying susceptibility to sleepiness-related outcomes. However, the intention of the present study is to assess OSA reactivation within a realistic clinical cohort.

Regarding sample size considerations, the calculations were based on previously reported standard deviations of MWT latencies from healthy and affected populations independently. The higher variability expected in affected participants was reflected in the larger required sample size for the OSA cohort. No manuscript changes were made, as the cited CPAP withdrawal model and sample size rationale were already addressed in the manuscript and supplementary material.

**Point 7:**
As the authors state, "it remains unclear whether EDS itself is consistently and measurably reactivated in this model, as no systematic data on its recurrence are currently available". Therefore, at the very least, the effect of CPAP withdrawal on subjective sleepiness in the patient population should be controlled (e.g., by appropriately modified ESS).

**Response:**
As partially addressed in the previous response, the present study uses an established CPAP withdrawal model previously shown to induce subjective and objective sleepiness-related changes. In the current study design, CPAP withdrawal effects are additionally controlled by repeated PSG measurements performed prior to each test day (lines 102–106). Furthermore, subjective sleepiness is systematically assessed using ESS scores for all participants and KSS ratings collected before and after each individual trial/run (lines 362–368).

**Point 8:**
The protocol lacks the assessment of basic vital signs (e.g., weight, height, blood pressure, heart rate) at trial start and after intervention, the latter for safety reasons and to control for effects on the bio-samples.

**Response:**
We thank the reviewer for this valuable suggestion. Information regarding participants’ weight, height, and heart rate is already collected prior to each PSG recording; however, this methodological detail was missing from the manuscript and has now been added (lines 297–300).

In addition, following the reviewer’s recommendation, blood pressure measurements were implemented during the screening visit (V1) and during each test day (V3, V4, V6, and V7) to improve physiological monitoring and control of potential influences on bio-sample-related outcomes. The corresponding adjustments were added to the manuscript (lines 292–293 and 304–306).

Regarding safety aspects, all participants undergo physician-led pre-screening prior to inclusion to ensure safe participation in the study procedures.

**Point 9:**
It is not specified how "sleep quality" and its night-to-night variability are to be assessed in the PSG recordings.

**Response:**
We thank the reviewer for this reasonable comment. The manuscript was revised and the corresponding section was expanded to further specify the assessment of sleep quality and night-to-night variability within the PSG recordings (lines 384–391).

**Point 10:**
The protocol names some inclusion and exclusion criteria. However, in the following text, it is mentioned that the participants in the patient group are referred from a partnering institution, and it is not clear how the referred patients are pre-selected by that institution.

**Response:**
The preselection of patients at the partnering institution includes assessment of CPAP therapy adherence as well as evaluation of comorbidities to ensure safe participation in the study. These procedures and eligibility considerations are described in the chapter “Intervention group (OSA patients)”.

**Point 11:**
The control group, which is relatively small, and the lack of any matching criteria reduces comparability with the clinical sample, even with statistical adaptation methods.

**Response:**
We agree that the healthy control group is not intended to represent a fully matched comparison cohort for the OSA patient population. The primary objective of the study is the comparison of the two wakefulness assessment methods within both healthy and OSA groups rather than direct between-group comparison. Nevertheless, the limited comparability between the healthy and clinical cohorts represents an important study limitation and was added to the limitations section of the manuscript (lines 736-740).

**Point 12:**
The use of medications with potential influence on sleepiness, and wake promoting agents (such as caffeine or smoking) in the participants is apparently not controlled or the control of these factors is not mentioned.

**Response:**
We thank the reviewer for this important comment. Wake-promoting substances and medications with potential influence on sleepiness are controlled within the study protocol; however, this information was insufficiently described in the manuscript. The corresponding section was revised and expanded to specify these control procedures (lines 321–326).

**Point 13:**
The large number of secondary outcomes also constitutes an issue. In the paper, many of these outcomes are listed in rather vague language, and no rationale is given why just these measures are selected, and how they are related to the basic question of the study, i.e., assessment of FTD.

**Response:**
We agree that the large number of secondary outcomes may appear broad in scope. These parameters are intended as exploratory outcomes and do not form the basis of the primary study hypothesis or a definitive predictive model for FTD assessment. Rather, they are included to explore potentially relevant multimodal markers of sleepiness and driving-related impairment based on previously published literature (lines 374-378).

To improve transparency, the manuscript was revised to better explain the selected exploratory measures and corresponding references were added. In addition, the limitations associated with multiplicity of secondary outcomes and exploratory model building are explicitly addressed in the limitations section of the manuscript.

**Point 14:**
A procedure to make original data available (with proper respect to the protection of patient identity) should be included in the paper.

**Response:**
The procedure for data availability and protection of participant confidentiality is described in the Data Availability section of the manuscript.
